# Progressive severe lung injury by zinc oxide nanoparticles; the role of Zn^2+ ^dissolution inside lysosomes

**DOI:** 10.1186/1743-8977-8-27

**Published:** 2011-09-06

**Authors:** Wan-Seob Cho, Rodger Duffin, Sarah EM Howie, Chris J Scotton, William AH Wallace, William MacNee, Mark Bradley, Ian L Megson, Ken Donaldson

**Affiliations:** 1 ELEGI Group, Centre for Inflammation Research, University of Edinburgh, Edinburgh, UK; 2 Immunology Group, Centre for Inflammation Research, University of Edinburgh, Edinburgh, UK; 3 Centre for Respiratory Research, University College London, London, UK; 4 Department of Pathology, Royal Infirmary of Edinburgh, University of Edinburgh, Edinburgh, UK; 5 School of Chemistry, West Mains Road, University of Edinburgh, Edinburgh, UK; 6 Free Radical Research Facility, UHI Department of Diabetes & Cardiovascular Science, Centre for Health Science, Inverness, UK

## Abstract

**Background:**

Large production volumes of zinc oxide nanoparticles (ZnONP) might be anticipated to pose risks, of accidental inhalation in occupational and even in consumer settings. Herein, we further investigated the pathological changes induced by ZnONP and their possible mechanism of action.

**Methods:**

Two doses of ZnONP (50 and 150 cm^2^/rat) were intratracheally instilled into the lungs of rats with assessments made at 24 h, 1 wk, and 4 wks after instillation to evaluate dose- and time-course responses. Assessments included bronchoalveolar lavage (BAL) fluid analysis, histological analysis, transmission electron microscopy, and IgE and IgA measurement in the serum and BAL fluid. To evaluate the mechanism, alternative ZnONP, ZnONP-free bronchoalveolar lavage exudate, and dissolved Zn^2+ ^(92.5 μg/rat) were also instilled to rats. Acridine orange staining was utilized in macrophages in culture to evaluate the lysosomal membrane destabilization by NP.

**Results:**

ZnONP induced eosinophilia, proliferation of airway epithelial cells, goblet cell hyperplasia, and pulmonary fibrosis. Bronchocentric interstitial pulmonary fibrosis at the chronic phase was associated with increased myofibroblast accumulation and transforming growth factor-β positivity. Serum IgE levels were up-regulated by ZnONP along with the eosinophilia whilst serum IgA levels were down-regulated by ZnONP. ZnONP are rapidly dissolved under acidic conditions (pH 4.5) whilst they remained intact around neutrality (pH 7.4). The instillation of dissolved Zn^2+ ^into rat lungs showed similar pathologies (eg., eosinophilia, bronchocentric interstitial fibrosis) as were elicited by ZnONP. Lysosomal stability was decreased and cell death resulted following treatment of macrophages with ZnONP *in vitro*.

**Conclusions:**

We hypothesise that rapid, pH-dependent dissolution of ZnONP inside of phagosomes is the main cause of ZnONP-induced diverse progressive severe lung injuries.

## Background

Zinc oxide nanoparticles (ZnONP) are utilised in many commercial products including cosmetics, paints, textiles, food additives, and personal hygiene products. Because ZnONP are translucent and highly effective in protection against ultraviolet A and B radiation, they are important ingredients of sunscreens and moisturizers [1]. ZnONP is widely used as an ingredient of paints and coating and finishing materials in products and buildings because they provide long-term protection from ultraviolet light [2]. ZnONP have also been used as a dietary supplement in human and livestock because Zinc can stimulate immune systems and act in an anti-inflammatory way [3,4]. ZnONP has external uses as antibacterial agents in ointments, lotions, mouthwashes, and surface coatings to prevent microorganism growth [5].

There are few toxicity reports on ZnONP despite their widespread use and potential for use in various applications. Toxicity studies of ZnONP have mainly focused on dermal toxicity, of relevance due to the inclusion of ZnONP within materials that are directly applied to skin. Penetration of ZnONP through normal skin was limited to the stratum corneum in porcine [6] and human models [7]. Exposure of human skin epithelial cells to ZnONP produced severe cytotoxicity accompanied by oxidative stress and genotoxicity [8]. Few studies have been reported concerning the *in vivo *toxicity of ZnONP although intratracheal instillation of ZnONP (50 - 70 nm) in Sprague-Dawley rats induced cytotoxicity and neutrophilic inflammation at 24 h after instillation [9].

Previously, we assessed the pulmonary inflammogenicity of a large panel of NP, including ZnONP and found that metal oxide NP elicited diverse patterns of inflammation with different cellular bases, at both the acute and chronic phase [10]. In the present study, we extended these experiments to specifically evaluate the mechanisms of eosinophilic inflammation and pulmonary fibrosis induced by ZnONP instillation.

## Methods

We briefly described materials and methods in the main text but the detailed methods were described in the Additional file 1.

### Characterization and dispersion of NP

ZnONP (10.7 ± 0.7 nm) were purchased from NanoScale Corporation (Manhattan, KS, USA) (Table 1). Surface area of ZnONP was determined with a Micromeritics TriStar 3000 (Bedfordshire, UK) by Escubed Ltd. (Leeds, UK). For dispersion of ZnONP, 5% of heat-inactivated rat serum (collected from the healthy female Wistar rat) was added to saline (Baxter, Deerfield, IL, USA) for final concentrations. The endotoxin levels of ZnONP at 300 cm^2^/ml were determined using a Limulus Amebocyte Lysate assay kit (Cambrex, Walkersville, MD, USA). The hydrodynamic size and zeta potential of ZnONP in PBS with 5% rat serum were assessed with a Brookhaven 90 plus (Holtsville, NY, USA) and Zetasizer-Nano ZS instrument (Malvern, Malvern Hills, UK), respectively.

**Table 1:** Characterization of nanoparticles.

Designated name		ZnONP	ZnONP_alt_	NiONP	TiO_2_NP
**Supplier**		**NanoScale Corp**.	**Nanostructure and Amorphous Materials Inc**.	**Nanostructure and Amorphous Materials Inc**.	**Nanostructure and Amorphous Materials Inc**.

Diameter (nm) examined by TEM		10.7 ± 0.7	137 ± 9.2	5.3 ± 0.4	30.5 ± 1.8
Surface area (m^2^/g)^a^		48.2	50	91.8	27.5
Mass (μg) per 150 cm^2 ^(high dose- rat)		310	-	163.5	545
Mass (μg) per 50 cm^2 ^(low dose- rat)		103	-	54.5	182
Mass (μg) per 15 cm^2 ^(high dose- mouse)		31	-	9.18	-
Zn^2+ ^(μg) equivalent to 50 cm^2 ^(rat study)		82.8	-	-	-
Endotoxin (EU/ml)^b^		ND	ND	ND	ND

Hydrodynamic size (nm) in	DW	2855 ± 773	4833 ± 625	1210 ± 471	787 ± 324
	PBS	3925 ± 715	5683 ± 510	2236 ± 407	1166 ± 344
	PBS (5% rat serum)	423 ± 24	282 ± 124	93 ± 4	119.1 ± 39.6
	PBS (5% mouse serum)	229 ± 187	-	83 ± 44	-

Zeta potential (mV) in	PBS (5% rat serum)	-27.1 ± 1.4	-25.9 ± 0.6	-26.0 ± 5.0	-28.5 ± 5.2
	PBS (5% mouse serum)	-18.9 ± 1.5	-	-21.4 ± 0.8	-

Values are mean ± S.D. from four independent experiments.

ND = not detectable.

^a^Determined by Escubed Ltd. (Leeds, UK).

^b^Lower detection limit 0.1 EU/ml.

DW, distilled water.

PBS, phosphate buffered saline.

### Durability of ZnONP

To evaluate the biopersistence of ZnONP *in vivo*, ZnONP were incubated with artificial lysosomal fluid (ALF) [11] and artificial pulmonary interstitial fluid (Gamble's solution) [12]. As a control particle, the rutile form of TiO_2_NP (30.5 ± 1.8 nm) was purchased from Nanostructure and Amorphous Materials Inc. (Houston, TX, USA) (Table 1). ALF (pH 4.5) and Gamble's solution (pH 7.4) were prepared as previously described [11,13]. ZnONP and TiO_2_NP were incubated with ALF or Gamble's solution at 5 mg/ml for 24 h at 37°C with gentle shaking. After 24 h, 50 mg of suspensions were centrifuged at 13 000 × *g *for 30 min and the supernatant discarded. After the final wash with distilled water (DW), pellets were air dried and resuspended in 5 ml of DW. NP suspensions were then weighed and calculated by subtraction of the weight of the container and the same volume of distilled water. There is potential for some error in this method due a small fraction of small NP that could remain in suspension after centrifugation and are lost on washing.

### Intratracheal instillation of ZnONP

Female Wistar rats (200 - 250 g) were humanely maintained and handled in accordance with the UK Home Office Animals Scientific Procedures Act. Intratracheal instillation was performed as previously described method [10]. ZnONP were instilled at a surface area dose of 50 or 150 cm^2^/rat, and 5% rat serum in saline was used as the vehicle control (*n *= 5 - 7 per group). Large agglomerates of ZnONP (diameter- 4,380 nm) in saline without rat serum also instilled at 150 cm^2 ^per rat to evaluate the effects of agglomeration on eosinophilia. We used surface area as a dose metric rather than mass because surface area has been known as a better descriptor of potential of NP to cause toxicity *in vitro *and *in vivo *[14]. To evaluate the time-course of the consequent inflammation, rats were sacrificed at 24 h, 1 wk, and 4 wks after instillation. Preparation of bronchoalveolar lavage (BAL) fluid and analysis for LDH and total protein was performed as previously described method [10].

### ELISA for pro-inflammatory mediators

Measurements of cytokines (TNF-α, IL-1β, IL-13, and TGF-β) and chemokines (MIP-2 and eotaxin) were performed in non-diluted BAL fluid following the manufacturer's instructions [IL-13 ELISA was obtained from Invitrogen (Camarillo, CA, USA) and other assays were from R&D Systems (Minneapolis, MN, USA)]. The detection limits of ELISA kit was as follow: TNF-α- 5 pg/ml; IL-1β- 5 pg/ml; TGF-β- 1.7 - 15.4 pg/ml; MIP-2- 0.5 - 2.7 pg/ml; eotaxin- 3 pg/ml; IL-13- 1.5 pg/ml.

### IgE and IgA ELISA in the serum and BAL fluid

To evaluate the serum immunoglobulin E (IgE) levels, ZnONP were instilled into rats (*n *= 4) at 150 cm^2 ^per rat and blood was taken via the tail vein at day 1 and week 1, 2, 3, and 4 after instillation. Serum was then collected and diluted 1 in 10 with PBS. Total serum IgE and IgA levels were determined using a rat IgE ELISA set and rat IgA ELISA set, respectively (all from BD Biosciences, Oxford, UK).

### Histological analysis (H&E, PSR, and PAS staining)

At each time point, histological analysis of lung tissues and picrosirius red (PSR) staining was performed as previously describe method [10]. For detection of goblet cells which contain mucin, periodic acid-Schiff (PAS) (Sigma-Aldrich) staining was performed according to standard methods. The quantitative image analysis of PAS-positive cells was performed using Image-Pro Plus (Media Cybernetics, MD, USA). PAS-positive signals in the airways were separately evaluated according to airway diameter; airways smaller than 1 mm were considered to be small airways or bronchioles and larger than 1 mm were considered to be a large airway or bronchi [15]. The total area of PAS-positive cells was divided by the total area of epithelial cells including basement membrane. The data were expressed as percentage of PAS-positive area versus total epithelial area.

### Immunohistochemistry for eotaxin, TGF-β, and α-SMA

Immunohistochemical staining for eotaxin, transforming growth factor-beta (TGF-β), and alpha-smooth muscle actin (α-SMA) was performed on lung sections. The detailed method for immunohistochemistry was described in Additional file 1.

### Transmission electron microscopy (TEM)

TEM was used to evaluate ultra-structural changes in the lungs induced by instillation of ZnONP. Lungs of vehicle control and ZnONP treated rats 4 wks after instillation were fixed with 1.5% glutaraldehyde in 0.1 M cacodylate buffer, stained *en bloc *with uranyl acetate, and embedded in epoxy resin. Ultra-thin (60 nm) sections were cut, stained with uranyl acetate and lead citrate, and examined with a TEM (JEM-1200EX II, JEOL, Tokyo, Japan).

### Instillation of alternative ZnONP

To evaluate whether the eosinophilic inflammation was induced by specific types of ZnONP, we instilled another type of ZnONP (designated ZnONP_alt_) into rats. ZnONP_alt _were purchased from Nanostructural and Amorphous Materials, Inc. (Houston, TX, USA) (Table 1). ZnONP_alt _were instilled into female Wistar rats at 310 μg per rat, which is the same mass of 150 cm^2 ^per rat as the ZnONP from NanoScale Corporation. Four rats were used for each treatment group. After 24 h, rats were euthanized and BAL collection and analysis was performed as described above.

### Instillation of ZnONP-free BAL extract to rats

To evaluate whether inflammatory mediators produced by ZnONP instillation can produce similar pathologies to that seen with ZnONP treatment, we extracted ZnONP-free BAL fluid and instilled this in rats. The detailed method was described in Additional file 1.

### Instillation of dissolved Zn^2+ ^to rats

To evaluate the effects of dissolved Zn^2+ ^in the acidic solution, 1 mg/ml of ZnONP in HCl-acidified saline were dissolved at a pH of 4.5. After 1 wk, ZnONP-free supernatant was collected by three rounds of centrifugation at 13000 × *g*. The supernatant was filtered three times through a 0.22 μm filter (Millipore, Cork, Ireland) to exclude possible bacterial contamination. The concentration of Zn^2+ ^of supernatant was measured by inductively coupled plasma-atomic emission spectrometry (ICP-OES) (Perkin Elmer Optima 5300 DV ICP-OES). The pH of the dissolved Zn^2+ ^was 6.5, which was less acidic than 0.9% saline (pH 5.5). Thereafter 92.5 and 277.5 μg of Zn^2+ ^were instilled intratracheally into rats and cytological and histological evaluation was performed at 24 h and 4 wks after instillation. The small discrepancy between the figure of 92.5 μg that was used in the experiment and the 82.8 μg that should have been used, was the result of an error where we originally calculated the equivalent Zn^2+ ^dose for ZnONP based on the zinc metal weight rather than zinc oxide weight.

### Aspiration of ZnONP into mice

To evaluate whether the eosinophilic inflammation was a species- and strain-specific phenomenon, ZnONP were aspirated into C57BL/6 and BALB/c mice. ZnONP were dispersed in 5% heat-inactivated mouse serum (collected from healthy C57BL/6 mice) to a dose of 150 cm^2^/ml and 100 μl (15 cm^2 ^ZnONP) were aspirated into the lungs of mice which were sacrificed 24 h later. As a control, NiONP (Table 1) known to cause acute neutrophilic inflammation [10] were aspirated at the same surface area dose. Four mice per each group were used for cytological evaluation. Eotaxin (R&D systems) and IL-13 (Invitrogen) levels were measured in the BAL samples as described above. TEM was also applied to evaluate the ultra-structural changes in the lung with the same method described above.

### Acridine orange staining

To evaluate the lysosomal membrane destabilization by NP, acridine orange staining was applied to THP-1 cells. Human monocytic cell line THP-1 was obtained from American Type Culture Collection (ATCC) and cultured at 37°C with 5% CO_2 _in RPMI containing 10% FBS, 2 mM L-glutamine (Life Technologies, Paisley, UK), 100 IU/ml penicillin, and 100 U/ml streptomycin (Life Technologies). THP-1 cells (1 × 10^6 ^cells/ml) were seeded to a μ-Dish^35 mm, high ^(Thistle Scientific Ltd., Glasgow, UK) and differentiated using 10 ng/ml of phorbol myristate acetate (PMA; Sigma-Aldrich) for 48 h. After activation, cells were washed three times with PBS and stained with 5 μg/ml acridine orange (Sigma-Aldrich) for 15 min. Cells were then washed three times with PBS and were treated with ZnONP (10 cm^2^/ml; 20 μg/ml) for 24 h. As a control, TiO_2_NP (10 cm^2^/ml; 36 μg/ml) were used. Cells were examined and photographed in a Leica SP5 confocal microscope (Leica Microsystems, Buckinghamshire, UK). Cytotoxicity was also measured using a lactated dehydrogenase assay kit according to the manufacturer's manual (Roche Diagnostics Ltd.).

### Statistical analysis

Data are expressed as mean ± S.D. and were analyzed with GraphPad InStat software (Version 3, GraphPad Software, Inc., La Jolla, CA). To compare each treatment group, one-way analysis of variance with Tukey's post hoc pairwise comparisons was applied. Student t-test was applied for comparison between vehicle control and ZnONP treatment group in C57BL/6 and BALB/c mice or ZnONP_alt _treatment group. We considered *p *< 0.05 to be statistically significant.

## Results

### Characterization of ZnONP

ZnONP showed a "hard agglomerates" which is not readily dispersed without any stresses (mechanical or sonication) in both PBS and DW (Table 1). However, when ZnONP were dispersed with serum protein, they showed a "soft agglomerates" which is readily dispersed without any stresses (mechanical or sonication) because of the protein corona on the surface of NP. The zeta potential of ZnONP in PBS was determined as -27.13 ± 1.36 mV. Endotoxin levels of ZnONP suspensions and rat serum were below the lower detection limit (0.1 EU/ml) whilst 5% mouse serum was calculated to contain 0.09 ± 0.02 EU/ml.

### Durability of ZnONP

**Figure 1:**
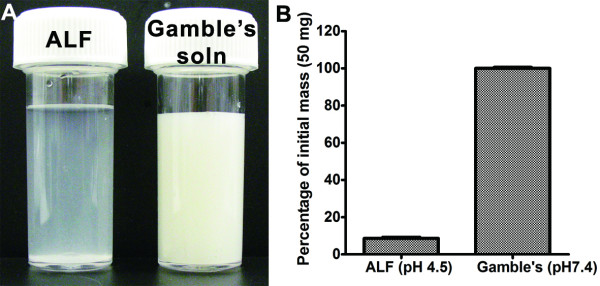
**Durability of ZnONP in artificial lysosomal fluid (ALF, pH 4.5) and artificial interstitial fluid (Gamble's solution, pH 7.4)**. ZnONP were incubated at 37°C for 24 h with gentle shaking. (A) Gross picture taken by digital camera. The photographs were taken before centrifugation. (B) Percentage of mass compared to initial mass (50 mg). Values are mean ± S.D. *n *= 3.

Around 90% of ZnONP mass was dissolved within 24 h by incubation with artificial lysosomal fluid at pH 4.5, whilst ZnONP in artificial interstitial fluid (Gamble's solution, pH 7.4) showed no dissolution (Figure 1). TiO_2_NP as a control showed no dissolution or loss of mass either in ALF or in Gamble's solution (data not shown). The presence of proteins/serum did not influence the durability of ZnONP (data not shown).

### Differential cell counts in the BAL fluid

**Figure 2:**
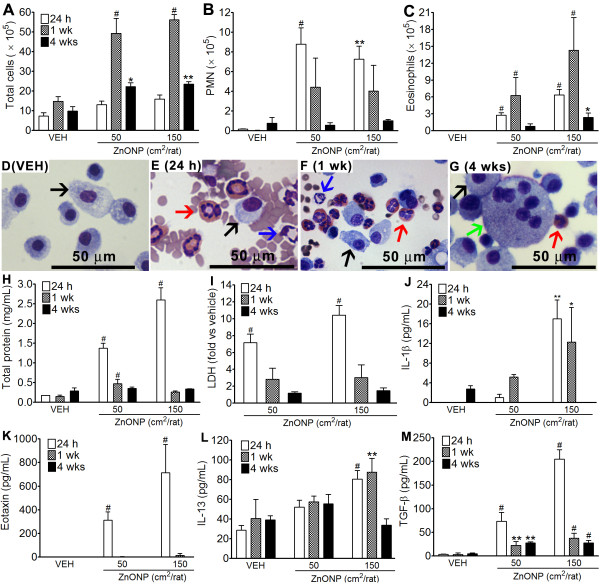
**Bronchoalveolar lavage (BAL) analysis 24 h, 1 wk, and 4 wks after instillation of ZnONP to rats**. (A - G), cytological analysis of BAL after instillation of ZnONP at 50 and 150 cm^2^/rat. (A), number of total cells; (B), number of PMN; (C), number of eosinophils. Representative BAL cell images of vehicle control (D) and ZnONP instillation (150 cm^2^) at 24 h (E), 1 wk (F), and 4 wks (G). Black arrows indicate alveolar macrophages; blue arrows indicate PMN; red arrows indicate eosinophils; green arrows indicate giant cells. (H and I), levels of total protein (H) and LDH (I) in the BAL from rats treated with ZnONP. Levels of LDH in BAL expressed as fold-change compared to vehicle control. (J - M), expression of inflammatory mediators in the BAL from rats instilled with ZnONP. (J), IL-1β; (K), eotaxin; (L), IL-13; (M), TGF-β. Values are mean ± S.D. *n *= 6 for 24 h and 4 wks groups and *n *= 4 for 1 wk groups. Significance versus vehicle control (VEH): * *p *< 0.05, ** *p *< 0.01, ^# ^*p *< 0.001.

Instillation of ZnONP produced significant increases in the total cell number at 1 wk and 4 wks, whilst at 24 h there was no significant change compared to control (Figure 2A). The number of polymorphonuclear leukocytes (PMN) was significantly increased at 24 h and returned to control levels thereafter (Figure 2B). The number of eosinophils was significantly increased at all time points and peaked at 1 wk after instillation (Figure 2C). The number of lymphocytes showed no significant changes in any treatment group. Representative images of BAL cells show that some giant cells were found at 4 wks after instillation (Figure 2G).

### Total protein and LDH in the BAL fluid

The levels of total protein and LDH in the BAL were significantly increased at 24 h after instillation and were comparable to controls thereafter except for total protein at 1 wk with low-dose ZnONP (Figures 2H and 2I).

### Pro-inflammatory cytokine levels in BAL fluid

The level of IL-1β was significantly increased 24 h and 1 wk after instillation of 150 cm^2 ^ZnONP (Figure 2J). Eotaxin expression was significantly increased only at 24 h with both doses whilst IL-13 was increased at 24 h and 1 wk with the 150 cm^2 ^dose only (Figures 2K and 2L). The concentrations of IL-1β, eotaxin, and IL-13 in the BAL were dose-related. The levels of TGF-β in the BAL peaked at 24 h and were still significantly increased at 1 and 4 wks after instillation. However, levels of TNF-α and MIP-2 showed no significant changes compared to controls (data not shown).

### IgE and IgA levels in the serum and BAL fluid

**Figure 3:**
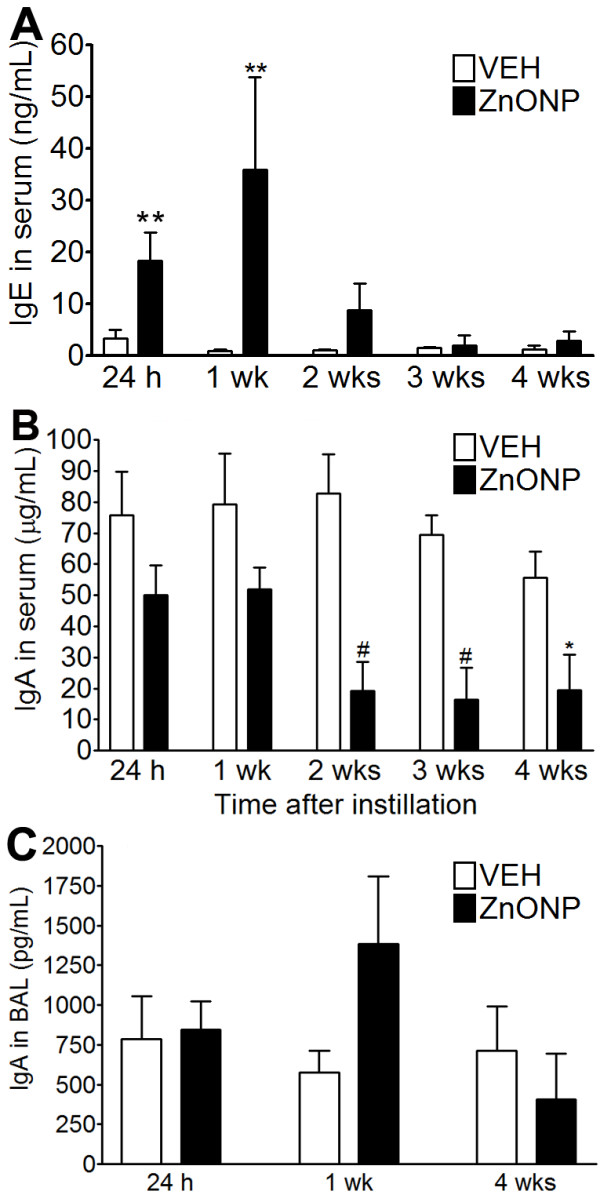
**IgE and IgA levels after instillation of ZnONP at 150 cm^2^/rat**. (A) IgE levels in serum; (B) IgA levels in serum; (C) IgA levels in BAL fluid. Values are mean ± S.D. *n *= 4 - 8 for each treatment group. Significance versus vehicle control: * *p *< 0.05, ** *p *< 0.01, ^# ^*p *< 0.001.

Serum IgE levels were transiently increased at 24 h and 1 wk following instillation of ZnONP instillation and were similar to controls thereafter (Figure 3A). However, IgE levels in the BAL were not significantly increased by any treatment (data not shown). IgA levels in the serum were significantly down-regulated 2, 3, and 4 wks after ZnONP instillation (Figure 3B). IgA levels in the BAL showed no significant changes compared to vehicle control (Figure 3C).

### Lung histopathology

ZnONP induced diverse pathological lung lesions both at the acute and chronic phase. The representative pathological lesions could be classified as eosinophilic inflammation, airway epithelial cell injury, regenerative proliferation, goblet cell hyperplasia, and pulmonary fibrosis with atelectasis (collapse of lung tissue affecting part or all of a lung).

#### Eosinophilic inflammation

**Figure 4:**
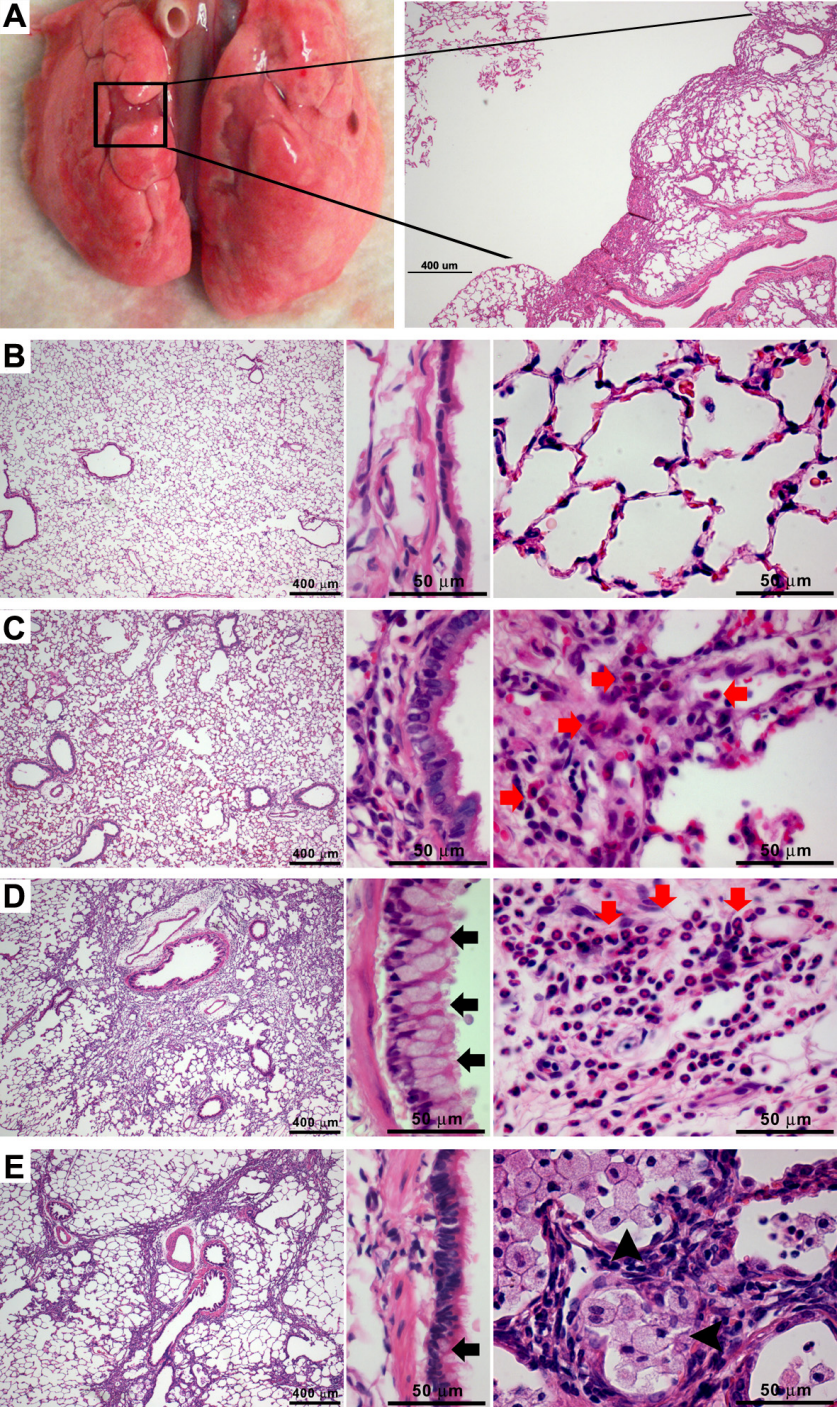
**Representative gross lesion and histology of lungs after instillation of ZnONP at 150 cm^2^/rat**. (A), lungs were contracted and collapsed by ZnONP treatment and this caused puckering of the lung surface. (B - E), each figure is composed of a low power view (left, 25×), high power view of bronchiolar epithelium (middle, 400×), and alveoli (right, 400×). (B) vehicle control at 24 h; (C) ZnONP at 24 h; (D) ZnONP at 1 wk; (E) ZnONP at 4 wks. Red arrows indicate eosinophils and arrowheads indicate foamy macrophages. Black arrows indicate goblet cells.

**Figure 5:**
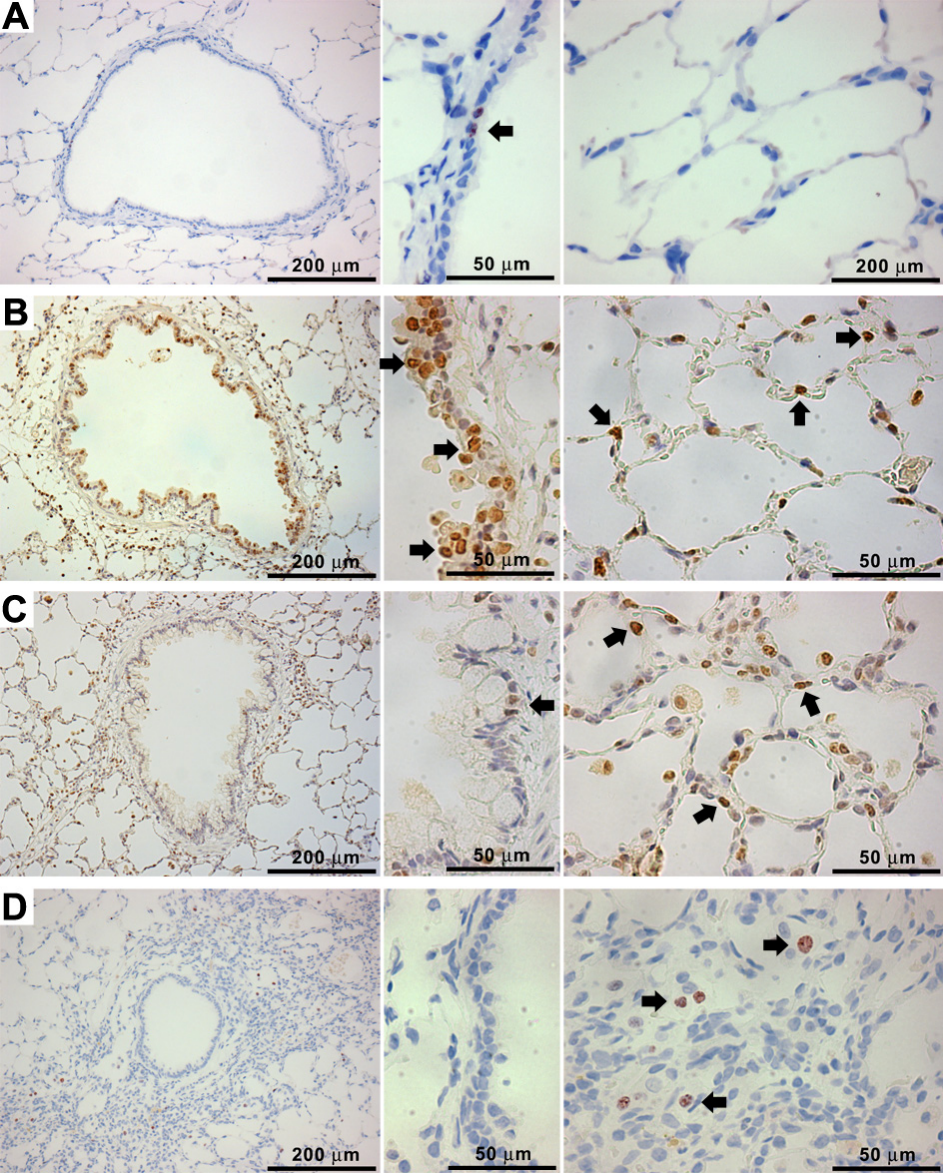
**Immunohistochemistry for Ki-67 in the lung tissues treated with ZnONP at 150 cm^2^/rat**. Each figure is composed of a low power view (left, 25×), high power view of bronchiolar epithelium (middle, 400×), and alveoli (right, 400×). (A) vehicle control at 24 h; (B) ZnONP at 24 h; (C) ZnONP at 1 wk; (D) ZnONP at 4 wks. Note that ZnONP increased the proliferating Ki-67 positive cells (arrow) in the bronchiolar epithelium and alveolar epithelium.

ZnONP induced severe eosinophilic inflammation in the lung tissues which was consistent with BAL fluid analysis (Figures 4C and 4D). Eosinophils were mainly present in interstitial areas including alveolar septum, peribronchial, peribronchiolar, and perivascular interstitium at all time points. Eosinophils in the alveoli were greatly increased at 1 wk, consistent with cytological analysis (Figure 4D). Lungs treated with ZnONP showed proliferation of type II cells 24 h and 1 wk after instillation (Figures 5B and 5C). At 4 wks after instillation, foamy macrophages had infiltrated into the alveoli and the eosinophilic inflammation was almost resolved (Figure 4E). Eotaxin, a specific chemoattractant for eosinophils, was strongly expressed at 24 h after ZnONP instillation (Additional file 2). The cells that stained most intensely for eotaxin were bronchial/bronchiolar epithelial cells although inflammatory cells were also positive for eotaxin to some extent.

#### Airway epithelial cell injury and goblet cell hyperplasia

**Figure 6:**
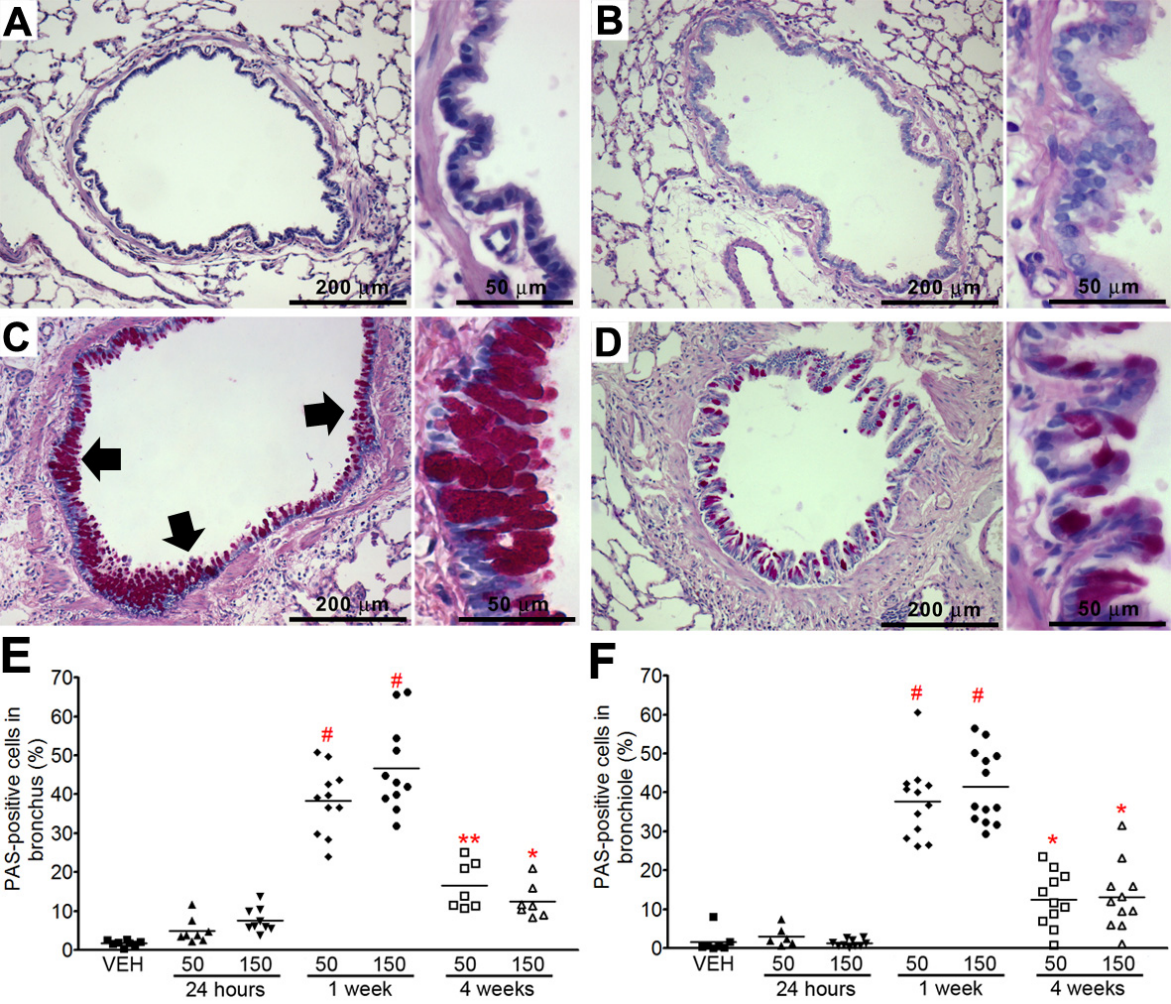
**PAS staining for goblet cells in the lung tissues treated with ZnONP at 150 cm^2^/rat**. Each panel is composed of low power view (left, 100×) and high power view of bronchiolar epithelium (right, 400×). (A) Vehicle control; (B) ZnONP at 24 h; (C) ZnONP at 1 wk; (D) ZnONP at 4 wks; Percentage of PAS-positive cells in the bronchus (> 1 mm in diameter) (E) and bronchiole (< 1 mm in diameter) (F). Goblet cells (arrow) peaked at 1 wk and still present 4 wks after ZnONP instillation. Each data point represents an independent bronchus or bronchiole in the lung tissues. Significance versus vehicle control: * *p *< 0.05, ** *p *< 0.01, ^# ^*p *< 0.001.

One of the most striking pathological effects of exposure to ZnONP was goblet cell hyperplasia and proliferation of airway epithelial cells including bronchial and bronchiolar epithelium. The normal bronchiolar epithelium is mainly composed of ciliated epithelial cells, goblet cells, Clara cells, and basal cells. In general, goblet cells are seen occasionally in PAS-stained section in the larger airways but are sparse in the bronchioles and absent from the terminal bronchioles where Clara cells predominate. Following ZnONP instillation, ciliated epithelial cells and basal cells became more basophilic and proliferation increased compared to vehicle control (Figure 4). The proliferation of airway epithelial cells was also confirmed by Ki-67 immunohistochemistry (Figure 5) showing that proliferation peaked at 24 h, was still apparent at 1 wk and had returned to control levels at 4 wks after instillation. Interestingly, the proliferation of airway epithelial cells was accompanied by striking hyperplasia of goblet cells which produce mucus (Figure 6). Goblet cells in the airway epithelium had undergone florid hyperplasia in both bronchi and bronchioles at 1 and 4 wks with the entire radius of the bronchiole being composed of goblet cells in some sections. At 1 wk, goblet cells could be found even in terminal bronchioles, but were not present in the transitional region between bronchiolar and alveolar tissue (Additional file 3). Whilst the goblet cell hyperplasia was transient at 1 wk, a modest excess of goblet cells were still evident at 4 wks.

#### Pulmonary fibrosis

**Figure 7:**
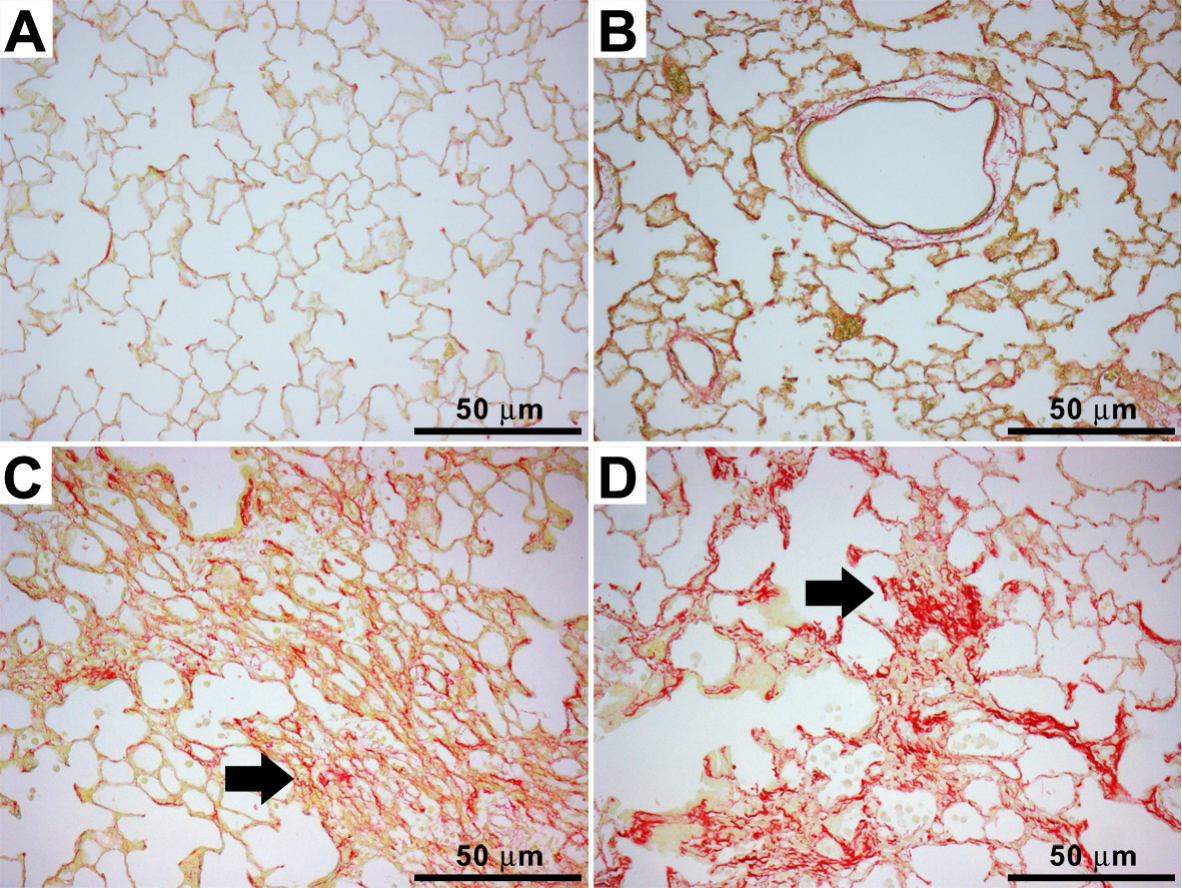
**Picrosirius red staining of the lung tissues treated with ZnONP at 150 cm^2^/rat**. (A) vehicle control; (B) ZnONP at 24 h; (C) ZnONP at 1 wk; (D) ZnONP at 4 wks. Note that collagen deposition (arrow) was increased 1 and 4 wks after instillation of ZnONP. Bar scale: 50μm.

**Figure 8:**
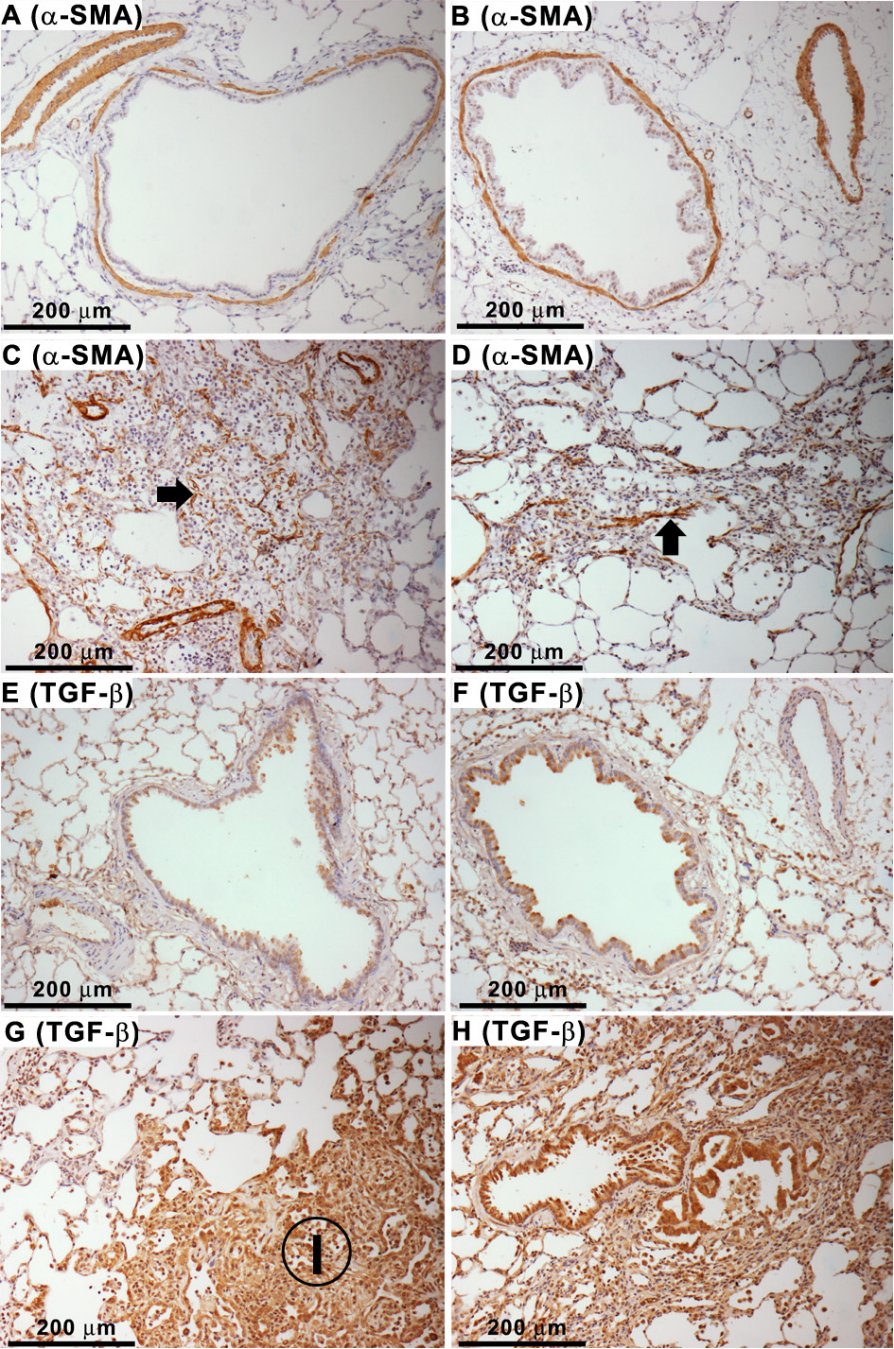
**Immunohistochemistry for α-SMA, and TGF-β in the lung tissues following treatment with ZnONP at 150 cm^2^/rat**. (A - D), α-SMA in the lungs. (A) vehicle control at 24 h; (B) ZnONP at 24 h; (C) ZnONP at 1 wk; (D) ZnONP at 4 wks. Note that α-SMA positive cells (dark brown staining) were seen in smooth muscle layer of airways and vessels of normal lung (A and B). In addition, they were seen profusely in the fibrotic lesions (arrow) at 1 (C) and 4 wks (D) after ZnONP instillation. (E - H), TGF-β in the lungs. (E) vehicle control at 24 h; (F) ZnONP at 24 h; (G) ZnONP at 1 wk; (H) ZnONP at 4 wks. Note that 'I' denotes the area of TGF-β positive fibrosis and the whole image of (H) was dominated by TGF-β positivity.

**Figure 9:**
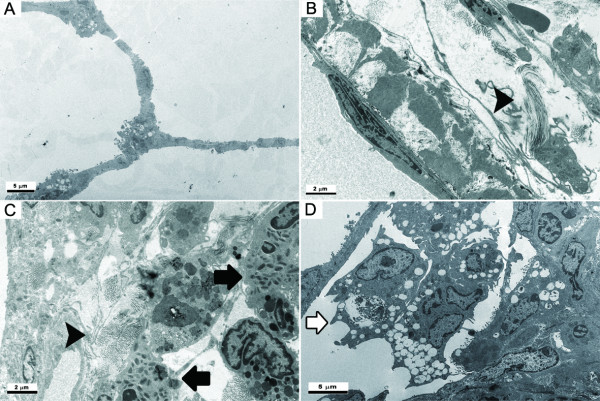
**Transmission electron microscopy images of lungs at 4 wks after instillation of ZnONP at 150 cm^2^/rat**. (A), vehicle control; (B - D), ZnONP treatment group. Bar scale: B and C = 2 μm; A and D = 5 μm. Collagen bundles (arrowhead) were found in the perivascular region (B) and alveolar interstitium (C) where they co-localized with eosinophils (black arrow) and PMN. Foamy macrophages (white arrow) were localized in the alveolar spaces.

Instillation of ZnONP induced interstitial and bronchocentric patterns of fibrosis, contraction and atelectasis of parenchymal lung tissue which was obvious as both gross lesions and in histological sections (Figure 4). The areas of fibrosis were evident especially at 1 and 4 wks after instillation, using picrosirius red (PSR) staining, (Figures 7C and 7D). The fibrotic and atelectatic lesions ran in bands through the parenchyma and where they met the pleura, caused puckering of the visceral pleural surface. Alpha-SMA, a myofibroblast marker, and transforming growth factor-β (TGF-β) were strongly expressed in these bands of fibrosis/atelectasis 1 and 4 wks after instillation (Figure 8). TEM examination showed large bundles of collagen, eosinophils, and neutrophils located in the interstitium and foamy vacuolated macrophages in the alveolar spaces in the lungs of ZnONP treated rats 4 wks after instillation (Figure 9). In addition to pulmonary fibrosis, the smooth muscle layer in the bronchi and bronchioles was thickened 1 and 4 wks after ZnONP instillation (Figure 4).

### Instillation of agglomerated ZnONP

Large poorly-dispersed agglomerates of ZnONP (diameter- 4,380 nm) produced around 91,000 eosinophils (1.3%) in the BAL whilst well-dispersed ZnONP (diameter- 242.9 nm) produced 595,000 eosinophils (36.7%) in the BAL (Additional file 4).

### Study with alternative ZnONP

**Figure 10:**
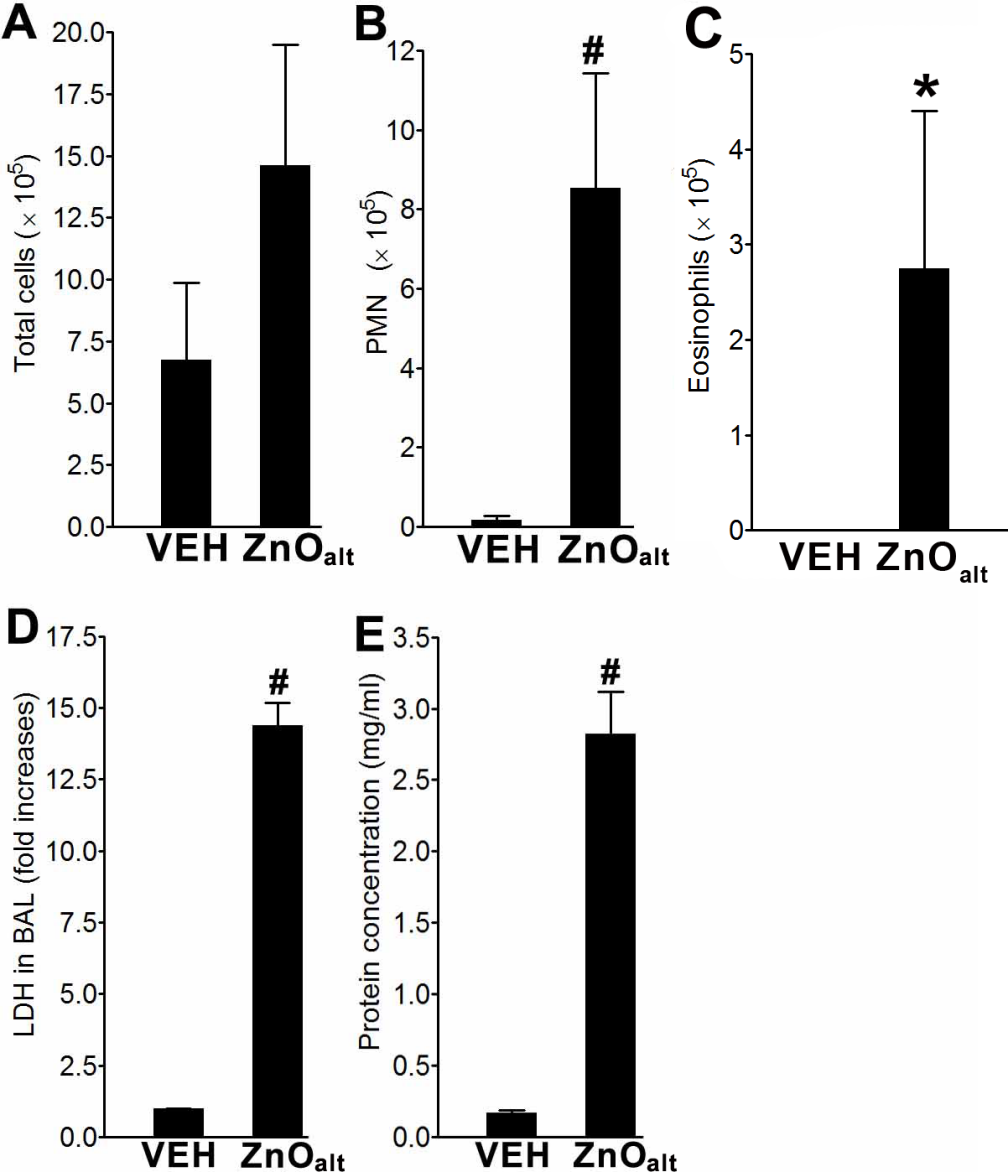
**Pulmonary toxicity of ZnONP_alt _at 24 h after instillation into lungs of rats**. (A-C), BAL cell analysis at 24 h after instillation of ZnONP_alt _to rats. (A) Number of total cells; (B) number of PMN; (C) number of eosinophils. Levels of LDH (D) and total protein (E) in the BAL fluid at 24 h after instillation of ZnONP_alt _to rats. Values are mean ± S.D. *n *= 4 for each treatment group. Significance versus vehicle control (VEH): * *p *< 0.05, ** *p *< 0.01, ^# ^*p *< 0.001.

To evaluate whether eosinophilic inflammation was a generic property of ZnONP, we instilled ZnONP obtained from an alternative commercial source that were slightly larger in size distribution (90 - 210 nm in size) into female Wistar rats. This ZnONP sample (designated ZnONP_alt_) significantly increased the number of PMN and eosinophils in the BAL following instillation (Figures 10B and 10C). In addition, ZnONP_alt _induced similar levels of LDH and total protein compared to ZnONP at the same mass dose (Figures 10E and 10F).

### Effects of NP-free BAL fluid on inflammation in rat lungs

NP-free BAL fluid collected 24 h after instillation of ZnONP into rats was instilled into naïve rat lungs. There was no inflammation at 1 and 4 wks after instillation of NP-free BAL fluid from ZnONP-exposed lungs (data not shown).

### Inflammatory pattern of dissolved Zn^2+ ^after instillation into rat lungs

**Figure 11:**
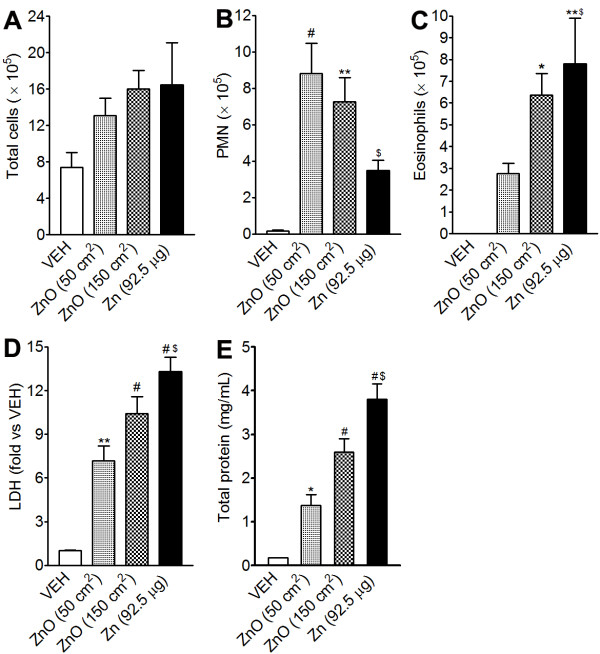
**Pulmonary toxicity of dissolved Zn^2+ ^at 24 h after instillation into lungs of rats**. Dissolved Zn^2+ ^in saline was instilled at 92.5 μg/rat. (A - C), cytological analysis of BAL cells. (A), number of total cells; (B), number of PMN; (C), number of eosinophils. Levels of LDH (D) and total protein (E) in the BAL at 24 h after instillation of ZnONP. Values are mean ± S.D. *n *= 4 for each treatment group. Significance versus vehicle control (VEH): * *p *< 0.05, ** *p *< 0.01, ^# ^*p *< 0.001. Zn^2+ ^(92.5 μg/rat) was compared with ZnONP at 50 cm^2^/rat: ^$ ^*p *< 0.05.

The high dose of soluble Zn^2+ ^(277.5 μg) but not the low dose of soluble Zn^2+ ^(92.5 μg) caused death of the rats because of overdose of highly toxic Zn^2+ ^in a single acute exposure at high dose rate. This is contrasting with the fact that similar dose of ZnONP was associated with 100% survival. Instillation of the low dose of soluble Zn^2+ ^caused severe eosinophilic inflammation and mild neutrophilic inflammation at 24 h after instillation (Figure 11). The number of eosinophils and levels of LDH and total protein of the low dose of Zn^2+ ^group were significantly higher but the number of PMN was significantly lower than those of ZnONP at 50 cm^2 ^(Figure 11). At 4 wks after instillation, the lungs showed very similar pathological lesions of ZnONP treatment including goblet cell hyperplasia, fibrosis, contraction, and atelectasis (Additional file 5).

### Inflammatory profile in the BAL after ZnONP aspiration into mice

**Figure 12:**
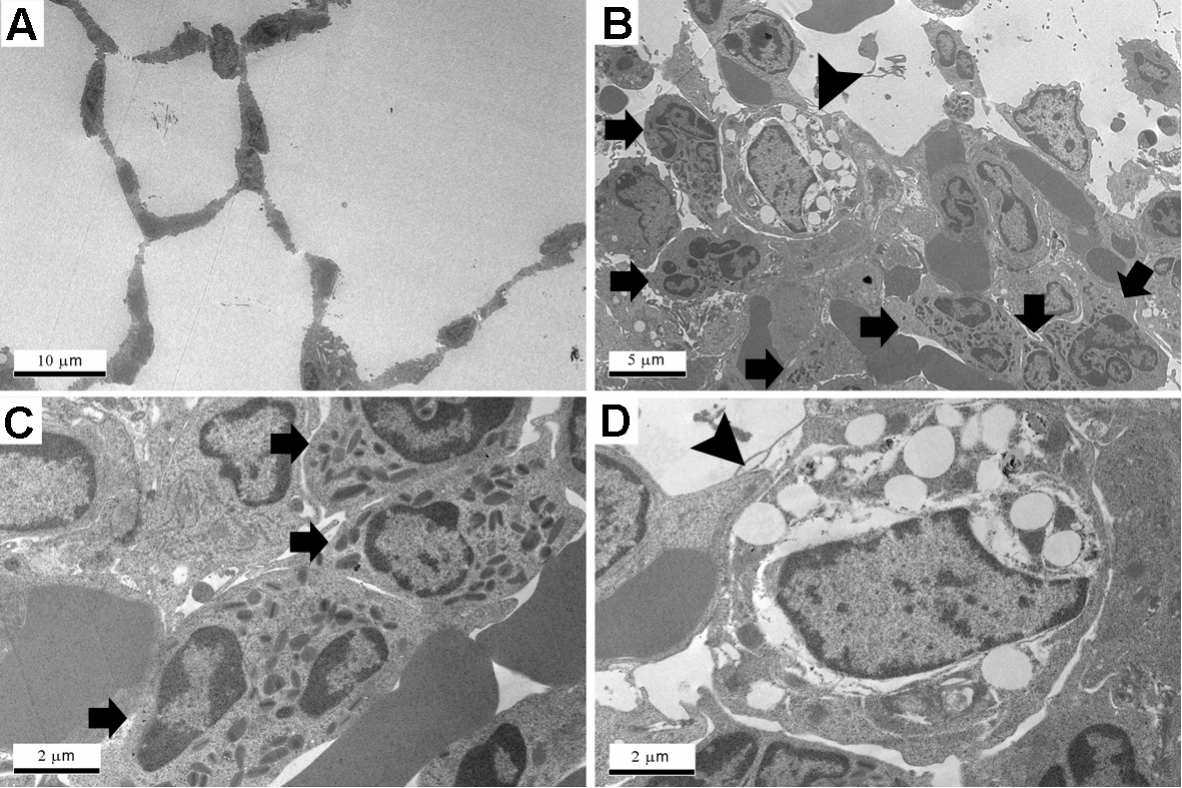
**Representative transmission electron microscopy images of lungs 24 h after instillation of ZnONP at 15 cm^2 ^to C57BL/6 mice**. (A), vehicle control; (B - D), ZnONP treatment group. Bar scale: A = 10 μm; B = 5 μm; C and D = 2 μm. Eosinophils (arrow) were infiltrated in the alveolar interstitium. Macrophage (arrowhead) was severely vacuolated.

ZnONP were inflammogenic in the lungs of C57BL/6 and BALB/c mice as evidenced by significantly increased numbers of PMN in the BAL at 24 h. However, neither strain showed eosinophilic inflammation at 24 h (Additional file 6). NiONP, a control particle, showed significant PMN recruitment (data not shown). Although no eosinophils were recruited into the BAL, the concentration of eotaxin and IL-13 in the BAL was significantly increased compared to vehicle control following ZnONP aspiration (Additional file 7). Although eosinophils were not detected in the BAL fluid, TEM analysis showed that the eosinophils were recruited in the alveolar interstitium (Figure 12). NiONP, a control particle, showed neutrophilic inflammation and neither eotaxin nor IL-13 showed significant changes compared to vehicle control (Additional files 6 and 7).

### Destabilization of lysosomes and cytotoxicity in macrophages exposed to ZnONP in vitro

**Figure 13:**
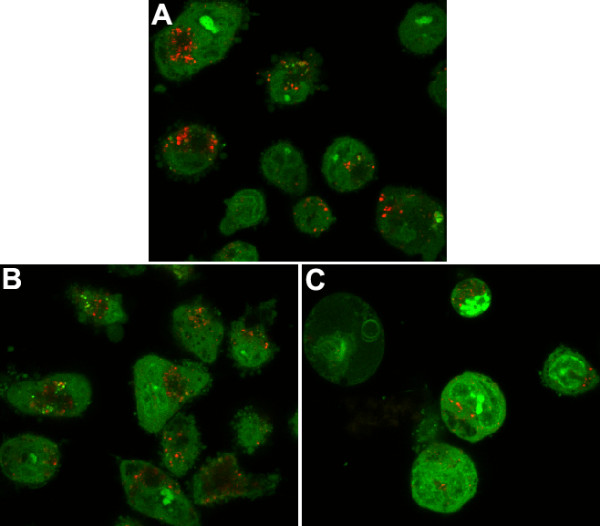
**Lysosomal destabilization by ZnONP in THP-1 cells**. Differentiated THP-1 cells by PMA (10 ng/ml) were stained with acridine orange for 15 min and cells were treated with NP for 24 h. (A), vehicle control; (B) TiO_2_NP at 10 cm^2^/ml (36.4 μg/ml); (C) ZnONP at 10 cm^2^/ml (20.6 μg/ml). Acridine orange dye aggregates inside of lysosome which showing red fluorescence. ZnONP showed less signals compared to vehicle control or TiO_2_NP.

Lysosomal staining with acridine orange, showing lysosomal stability, waned following ZnONP treatment compared to vehicle control and this was accompanied by loss of viability (Additional file 8). In contrast, vehicle control and TiO_2_NP-exposed macrophages showed high acridine orange fluorescence intensity confirming that these cells had intact lysosomes (Figure 13) and this was reflected in lack of cell death in the exposed macrophages (Additional file 8).

## Discussion

In previous studies we found that zinc oxide nanoparticles (ZnONP) were highly fibrogenic and caused an eosinophil exudate into the BAL, a finding that is highly unusual and possibly unique following particle exposure [10]. The current paper set out to determine the likely mechanism of the effects of ZnONP. This paper is notable by its lack of systematic inclusion of benchmark or control particles. The reason is that we have already published extensive findings on the ZnONP response compared to other metal oxides and control NP [10]. The aim of the present paper was solely to investigate the detail of the ZnONP-induced response with a view to better understanding the mechanism whereby they cause such severe pathological effects in rat lungs following a single intratracheal instillation.

In this study, ZnONP were well dispersed with serum protein. Stability of NP depends on a balance between attractive and repulsive forces between particles [16]. Incubation of NP with serum protein forms a protein corona which acts as steric stabilizer preventing agglomeration [17]. When NP deposit in the lung, surfactant proteins and lipids are adsorbed onto the NP forming a lipoprotein corona. Therefore, intratracheal instillation of NP dispersed with serum protein partially mimics the interaction of NP with the lung surface environment. To minimize xenogeneic effects, the serum protein from the same strain animals as those used in the experiments was used as a dispersion medium.

All NP in this study showed negative charge by zeta potential measurement because of the negatively charged protein corona which is the actual charge that is encountered by cells [18]. The recognition by phagocytes facilitates phagocytosis which ingests NP into phagosomes which are then acidified by phagosome/lysosomes fusion. Because ZnONP showed fast dissolution in acidic solution, they are not likely to persistent in the acid milieu of the phagolysosomes. Therefore the effect of surface charge is likely to be less important for high-solubility NP although insoluble positively charged NP are more toxic than neutral or negatively charged NP [19].

ZnONP induced pulmonary eosinophilia from 24 h to 4 wks after a single intratracheal instillation without any prior sensitisation process. There are published studies showing eosinophilic inflammation in animals following various sensitisation procedures; particles such as TiO_2_NP [20] and ambient particulate matter [21] have been implicated in enhancing development of the murine model of allergic asthma. In addition, instillation or intravenous injection of Sephadex beads (complex of cross-linked dextran polymers; diameter: 20 - 50 μm) also reported to cause eosinophilic inflammation in the lung [22]. However, the eosinophilia caused by Sephadex beads may be produced by their extremely large size because ultrasonication of this particle produced only a transient neutrophilic inflammation [22]. In addition, the Sephadex beads are polymer particles which are fundamentally different from ZnONP used in this study. NP-free BAL exudates collected 24 h after rats were instilled intratracheally with ZnONP at 150 cm^2 ^did not produce any inflammatory reaction either at 1 wk or 4 wks. Considering that eosinophilia peaked 1 wk after instillation and pulmonary fibrosis was mature 4 wks after instillation, the lack of inflammation caused by NP-free BAL exudate instillation suggest that BAL fluid might be too diluted to produce the pathologies caused by ZnONP instillation.

In mice, ZnONP aspiration caused eosinophilia but eosinophils were not found in the alveoli (BAL fluid) but were present in the alveolar interstitium. The interstitial type of eosinophilia was consistent with the elevated levels of eotaxin and IL-13 in the BAL. Eosinophils in the interstitium are as likely to cause tissue injury as eosinophils in the bronchoalveolar space, if not more so [23].

We undertook a number of assays to investigate the mechanism of the complex and severe pathological syndrome seen following exposure to ZnONP. In the conventional rodent asthma model, recruited eosinophils are associated with airway remodelling including peribronchial fibrosis, smooth muscle hyperplasia, and mucus secretion with the involvement of eotaxin and IL-13 [24,25]. In the ZnONP model we found that eotaxin and IL-13 were produced early in rats and mice exposed to ZnONP and these are key mediators of eosinophil recruitment [25,26]. IL-13 is especially involved in the regulation of eosinophil infiltration, IgE synthesis, goblet cell hyperplasia, mucus hypersecretion, and sub-epithelial fibrosis in asthma [25,27]. Therefore, IL-13 provoked by ZnONP might exert an important role on the wide spectrum of pathological effects seen here with ZnONP exposure.

Although the main inflammatory cells induced by ZnONP were eosinophils, PMN were also recruited during the acute phase. PMN have been regarded as the representative acute inflammatory cells playing a role in particle effects and their recruitment is highly correlated with the surface area dose of low-toxicity, low-solubility particles [28] and toxic particle such as crystalline silica [14]. The pro-inflammatory cytokine recruiting PMN into ZnONP-exposed lungs was most likely IL-1β which is known to induce neutrophilic inflammation in the lung [29]. However, PMN recruitment by ZnONP was confined to the 24 h time-point whilst significant IL-1β in BAL continued to the 1 week time-point at the highest dose.

Intratracheal instillation of ZnONP induced massive proliferation of airway epithelial cells and goblet cell hyperplasia. ZnONP were reported to be very cytotoxic to BEAS-2B cells *in vitro *by generating reactive oxygen species [30]. In the present study, ZnONP dramatically increased the levels of LDH and total protein in the BAL at the acute phase indicating cell death and increased vascular permeability, respectively. Therefore, the proliferation of airway epithelial cells likely represents a regenerative response to the cytotoxicity induced by ZnONP. Proliferation of airway epithelial cells led to large-scale goblet cell hyperplasia. Goblet cell hyperplasia was most pronounced at 1 wk and had waned by 4 wks after instillation of ZnONP. Goblet cell hyperplasia plays an important role in protecting the airway from damage due to inhaled particles [31]. The resulting increase in mucus flow traps inhaled particles and removes them from the airways by muco-ciliary clearance. In addition, goblet cells can be progenitors of ciliated cells to maintain mucus flow [32] and hyperplasia of goblet cells is reversible on cessation of administration [31]. Hypersecretion of mucus by goblet cell hyperplasia is also a feature of airway injury including exposure to cigarette smoke, and sulphur dioxide, and in asthma where it can contribute to obstruction of airways [33]. TGF-β is known to enhance goblet cell hyperplasia and mucus hyper-secretion in mice through an NF-κB-dependent mechanism [24]. In this study, we found strong immunostaining for TGF-β at all time-points, but particularly at 1 and 4 wks after instillation when goblet cell hyperplasia was most pronounced. Consistent with the immunostaining results, total TGF-β concentration in the BAL was also increased at all time-points.

Massive pulmonary fibrosis with contraction and atelectasis was also induced by ZnONP instillation. Following ZnONP instillation collagen fibres, determined by PSR staining, were increased from 1 wk and were more marked at 4 wks. At 4 wks, TEM images showed that large swathes of collagen fibres were primarily located in the perivascular and alveolar interstitium co-localized with eosinophils. Myofibroblasts, the major source of extracellular matrix proteins and contractile forces during fibrogenesis [34], were also seen in the contracted and fibrotic lung lesions at 1 and 4 wks after treatment. TGF-β also is known to cause airway remodelling including peribronchial fibrosis and smooth muscle hyperplasia [24].

Instillation of ZnONP increased IgE levels in the serum but not in the BAL. Multi-walled carbon nanotubes [35], diesel exhaust particles [36], and ultrafine carbon black [37] have all been reported to increase serum IgE levels by an adjuvant-like mechanism in murine asthma models. Surprisingly, compared to previous studies, the increase in IgE levels by ZnONP were induced by just a single instillation without any sensitization process. The levels of IgA in the serum were decreased compared to vehicle control. Although there was no statistical significance, IgA levels in the BAL showed an increasing trend at 1 wk after instillation of ZnONP. Increases in mucosal secretory IgA were present in the BAL, and this might explain the decrease of IgA in serum if BAL IgA was derived from the vascular space. The increased IgA levels in the BAL might act be anti-inflammatory in inflamed lung [38]. TGF-β is also known to induce IgA isotype expression by activating Smad3/4 complex translocation into the nucleus [39].

Our data on ZnONP durability in acid and neutral conditions suggests a mechanism for pathogenicity of ZnONP. ZnONP were stable at neutral pH or saline but very rapidly dissolved in the acidic artificial lysosomal fluid (pH 4.5). Thus ZnONP in the approximately neutral surfactant fluid or in the cytosol might be persistent. However, when ZnONP are internalized into the acid environment of the lysosome, they will be rapidly dissolved producing a high local concentration of Zn^2+ ^ions. Zn^2+ ^is one of the essential elements in cell homeostasis and remains in a bound form inside cells because free Zn^2+ ^is very reactive and cytotoxic [40]. The acute increase in free Zn^2+ ^levels may damage lysosomes, allowing the contents to escape into the cytoplasm where they may damage other organelles leading to cell death [41,42]. We suggest therefore that Zn^2+ ^released from the phagolysosomes of dead or damaged cells is the source of the Zn^2+ ^after ZnONP uptake in the lungs. The interaction of Zn^2+ ^with cells generates oxidative stress and finally triggers cell death signalling cascades [41,43]. When ZnONP were added to activated THP-1 cells (a differentiated macrophage cell line), lysosomes were destabilised by a mechanism which seems likely to involve dissolution of ZnONP under the acid condition of the lysosomes. Unlike ZnONP, TiO_2_NP showed no dissolution in acid or neutral conditions and when incubated with macrophages the fluorescence intensity of lysosomes was not reduced. The loss of lysosomal integrity induced by ZnONP was accompanied by cell death. A role for dissolved Zn^2+ ^in the toxic mechanism of ZnONP is further supported by Muller et al. [44] who reported that ZnO nanowires were rapidly dissolved in the acidic pH of lysosomes causing structural changes in mitochondria and cell death including necrosis and apoptosis [44].

Instillation of dissolved Zn^2+ ^from ZnONP treated under acid conditions in saline, produced eosinophilia 24 h after instillation. Moreover, the toxicity of soluble Zn^2+ ^was much greater than similar mass dose of ZnONP based on mortality, number of eosinophils, and levels of LDH and total protein. The higher number of eosinophils by ZnONP than that of ZnONP_alt _might be due to the smaller primary particle size and their wider distribution inside of the lung. The chronic lung lesions caused by Zn^2+ ^instillation were similar to those induced by ZnONP in terms of bronchocentric interstitial pulmonary fibrosis, goblet cell hyperplasia, and atelectasis.

There are many inhalation/instillation studies using zinc compounds although few papers on nanoparticles. No papers reported eosinophilia on exposure to zinc compounds except for our previous papers using ZnONP [10,45] and one human case report with zinc oxide [46]. The possible reason for this discrepancy is (1) species and strain differences, (2) location of eosinophils infiltration to the interstitium or airspaces, (3) dose, (4) polydispersity of particles, and (5) durability of particles. In particular the dispersed NP size might be important as we have shown by comparing well-dispersed and agglomerated NP which might specifically address the role of ZnONP agglomerate size. We noted that large poorly-dispersed agglomerates of ZnONP recruited much less number and percent eosinophils (1.3%) compared to well-dispersed ZnONP (37.7%). Based on this data we also conclude that micron-sized particles would not cause substantial eosinophilia in BAL following instillation. This finding is consistent with our previous study which showed no eosinophilic inflammation with ZnONP_alt _(137 nm) using same strain of rats and instillation technique used in here [47]. In addition, other previous publications also showed no eosinophilic inflammation by nano-size ZnO (50 -70 nm) or micrometer-size ZnO (< 1000 nm) [9,48]. These previous studies instilled ZnO without any dispersion which implies agglomeration into much larger particles whose compartmentation in the lung might differ from singlet NP or small agglomerates. Therefore, the eosinophilic inflammation by ZnONP may be elicited only by exposure to ZnONP well dispersed and at high doses.

ZnONP-induced eosinophilia, fibrosis, and goblet cell hyperplasia mediated by the soluble Zn^2+ ^is to our knowledge a novel finding in rat lungs. Interestingly, one epidemiological study showed that the level of Zn^2+ ^in ambient particulate matter was associated with asthma morbidity in USA [49]. Therefore, ZnONP pose a unique and substantial hazard to the lungs and hygiene precautions and control of airborne exposure should be instituted in any situation with the potential for exposure in order to reduce the risks of the kinds of lung pathology described here.

## Conclusion

**Figure 14:**
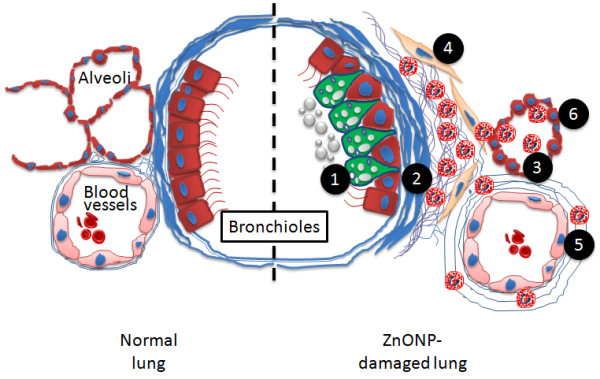
**Diagram of lung injury caused by ZnONP instillation**. ZnONP induced (1) goblet cell hyperplasia, (2) thickening of bronchiolar smooth muscle layer, (3) eosinophilia, (4) fibrosis with increased numbers of myofibroblasts (yellow) and collagen (thin blue fibres), (5) perivascular edema, and (6) type II cell hyperplasia.

Figure 14 summarises the diverse pathological changes (eosinophilia, airway epithelial cell injury, regenerative goblet cell hyperplasia, bronchocentric pulmonary fibrosis, and atelectasis) induced by a single installation of ZnONP. Although more studies are required, our data suggest that a single high exposure of ZnONP produced eosinophil influx as well as severe fibrosis and airway epithelial injury. The main cause of these effects appears to be the dissolution of ZnONP in the acid milieu inside phagosomes. This study suggests that dissolution of ZnONP to ions in the acid environment of the lysosomes causes lysosomal destabilisation and cell death. The resultant widespread cell death along the path followed by the instilled dose is likely a key factor in the severe cell death and subsequent pathogenicity seen. Exposure to ZnONP should be strictly regulated in occupational/consumer setting to minimise the likelihood of such severe adverse effects occurring.

## Competing interests

The authors declare that they have no competing interests.

## Authors' contributions

WSC, WMacN, ILM, MB, SEMH and KD provided key intellectual input culminating in the conception and design of these studies and aided in the writing of this manuscript. The studies were carried out by WSC and RD who also contributed to the writing of the manuscript. CJS provided expertise and materials for immunohistochemistry and WAHW interpreted the pathological slides and both contributed to the writing of the manuscript. All authors read and approved the final manuscript.

## Supplementary Material

Additional file 1 **Detailed materials and methods**. All detailed materials and methods were described. Click here for file

Additional file 2 **Immunohistochemistry for eotaxin in the lung tissues 24 h after instillation of ZnONP at 150 cm^2^/rat**. (A), vehicle control; (B and C), ZnONP treatment; (D) serially sectioned H&E staining of (C). (B) is imaged under higher magnification at 400× in (C). Note that eotaxin was strongly positive in the bronchial epithelial cells (arrow) and inflammatory cells (arrowhead). Click here for file

Additional file 3 **PAS staining for goblet cells in the lung tissues 1 wk after instillation of ZnONP at 150 cm^2 ^per rat**. Goblet cells were found even in terminal bronchioles (arrow) but were not present in the transitional region between bronchiolar and alveolar tissue. Click here for file

Additional file 4 **The effects of hydrodynamic size of ZnONP on the eosinophilia (*n *= 4)**. This file contains the number of eosinophils in the BAL after instillation of well-dispersed or highly agglomerated ZnONP. Click here for file

Additional file 5 **Representative lung lesion 4 wks after instillation of Zn(II) at 92.5 μg per rat**. (A) The lungs showed fibrosis, contraction, atelectasis, and (B) goblet cell hyperplasia. Click here for file

Additional file 6 **Pulmonary toxicity of ZnONP at 24 h after aspiration into lungs of C57BL/6 (A - C) or BALB/c (D - F) mice**. (A, D), number of total cells; (B, E), number of PMN; (C, F), number of eosinophils. Values are mean ± S.D. *n *= 4 for each treatment group. Significance versus vehicle control (VEH): * *p *< 0.05, ** *p *< 0.01, ^# ^*p *< 0.001. Click here for file

Additional file 7 **Expression of eotaxin and IL-13 in the BAL from mice 24 h after aspiration of ZnONP or NiONP at 15 cm^2 ^per mouse**. (A), eotaxin; (B), IL-13. Values are mean ± S.D. *n *= 4 for each treatment group. Significance versus vehicle control (VEH): * *p *< 0.05, ** *p *< 0.01, ^# ^*p *< 0.001. NS, not significant. Click here for file

Additional file 8 **Cytotoxicity of THP-1 cells after exposure to NP for 24 h, measured as percentage compared to complete lysis (Triton X-100)**. THP-1 cells were differentiated by treatment with PMA (10 ng/ml) for 48 h and LDH levels were measured 24 h after NP treatment. Values are mean ± S.D. *n *= 4 for each treatment group. Significance versus vehicle control (VEH): ^# ^*p *< 0.001. Click here for file
