# Supplementary material for: Progressive severe lung injury by zinc oxide nanoparticles; the role of Zn2+ dissolution inside lysosomes
Source: Part Fibre Toxicol. 2011 Sep 6;8:27. doi: 10.1186/1743-8977-8-27 (PMC3179432; doi:10.1186/1743-8977-8-27)
Supplement: Additional file 1 — Detailed materials and methods. All detailed materials and methods were described. [file 1743-8977-8-27-S1.DOC]

# Methods

***Characterization and dispersion of NP***

ZnONP (10.7 ± 0.7 nm) were purchased from NanoScale Corporation (Manhattan, KS, USA) (Table 1). Surface area of ZnONP was determined with a Micromeritics TriStar 3000 (Bedfordshire, UK) by Escubed Ltd. (Leeds, UK). Briefly, a weighed quantity of each sample was introduced to the sample tubes, degassed for 10 min under nitrogen at room temperature before being heated to 150 °C for 2 h, again under nitrogen. The samples were then allowed to cool before being reweighed and assessed for surface area. The gases used for the analysis were nitrogen and helium. N_2_ adsorption-desorption isotherms were conducted at 77 K on a Micromeritics Tristar 3000 analyzer. The BET surface areas were concluded using adsorption data. For dispersion of ZnONP, 5% of heat-inactivated rat serum (collected from the healthy female Wistar rat) was added to saline (Baxter, Deerfield, IL, USA) for final concentrations. The endotoxin levels of ZnONP at 300 cm^2^/ml and serum for dispersion (collected from healthy rat and mouse) were determined using a QCL-1000 Chromogenic End-Point Limulus Amebocyte Lysate assay kit (Cambrex, Walkersville, MD, USA). The hydrodynamic size and zeta potential of ZnONP in PBS with 5% rat serum were assessed with a Brookhaven 90 plus (Holtsville, NY, USA) and Zetasizer-Nano ZS instrument (Malvern, Malvern Hills, UK), respectively.

***Durability of ZnONP***

To evaluate the biopersistence of ZnONP *in vivo*, ZnONP were incubated with artificial lysosomal fluid (ALF) [1] and artificial pulmonary interstitial fluid (Gamble’s solution) [2]. As a control particle, the rutile form of TiO_2_NP (30.5 ± 1.8 nm) was purchased from Nanostructure and Amorphous Materials Inc. (Houston, TX, USA) (Table 1). ALF (pH 4.5) and Gamble’s solution (pH 7.4) were prepared as previously described [1, 3]. ZnONP and TiO_2_NP were incubated with ALF or Gamble’s solution at 5 mg/ml for 24 h at 37 °C with gentle shaking. After 24 h, 50 mg of suspensions were centrifuged at 13 000 × *g* for 30 min and the supernatant discarded. To minimize the interference of precipitated salt crystals in the assessment of mass, pellets of NP were washed three times with 5 ml of distilled water (DW) and centrifuged at 13 000 × *g* for 30 min. After the final wash, pellets were air dried and resuspended in 5 ml of DW. NP suspensions were then weighed and calculated by subtraction of the weight of the container and the same volume of distilled water.

***Intratracheal instillation of ZnONP***

Female Wistar rats (200 - 250 g) were humanely maintained and handled in accordance with the UK Home Office Animals Scientific Procedures Act. Rats were anesthetized with isofluorane and the trachea was cannulated with the aid of a laryngoscope. ZnONP were then intratracheally instilled at a surface area dose of 50 or 150 cm^2^/rat, and 5% rat serum in saline was used as the vehicle control (*n* = 5 - 7 per group). We used surface area as a dose metric rather than mass because surface area has been known as a better descriptor of potential of NP to cause toxicity *in vitro* and *in vivo* [4]. The mass doses of ZnONP for 50 and 150 cm^2^/rat were determined as approximately 0.5 and 1.5 mg/kg body weight. To evaluate the time-course of the consequent inflammation, rats were sacrificed at 24 h, 1 wk, and 4 wks after instillation.

***Cytological analysis of BAL***

At each time point, the lungs were lavaged 4 times with 8 ml of saline and first 8ml of bronchoalveolar lavage (BAL) fluid was kept separately for assays including lactate dehydrogenase (LDH), total protein, and inflammatory mediators. Cell pellets from the 4 lavages of each animal were pooled for cytological analysis. BAL fluid was centrifuged at 250 × *g* for 5 min and cell pellets were resuspended in 1 ml of PBS. Total cell number was counted by a nucleocounter (Chemometec, Allerod, Denmark) and 10 000 cells were attached by cytospin at 15 × *g* for 5 min. The slides were stained using Diff-Quik dyes (Raymond Lamb, Eastbourne, UK). Around 300 – 500 cells per each slide were differentially counted under a light microscope.

***BAL fluid analysis (LDH, total protein, inflammatory mediators)***

The levels of LDH (Roche Diagnostics Ltd., West Sussex, UK) and total protein (bicinchoninic acid assay; Sigma-Aldrich, Gillingham, Dorset, UK) in the BAL were measured according to the manufacturer’s instructions. Measurements of cytokines (TNF-α, IL-1β, IL-13, and TGF-β) and chemokines (MIP-2 and eotaxin) were performed in non-diluted BAL fluid following the manufacturer’s instructions [IL-13 ELISA was obtained from Invitrogen (Camarillo, CA, USA) and other assays were from R&D Systems (Minneapolis, MN, USA)].

***IgE and IgA ELISA in the serum and BAL fluid***

To evaluate the serum immunoglobulin E (IgE) levels, ZnONP were instilled into rats (*n* = 4) at 150 cm^2^ per rat and blood was taken via the tail vein at day 1 and week 1, 2, 3, and 4 after instillation. Serum was then collected and diluted 1 in 10 with PBS. To minimize the interference of IgG, serum was pre-incubated with Protein G beads (GE Healthcare UK Ltd, Buckinghamshire, UK) for 1 h at room temperature to remove it. Total serum IgE and IgA levels were determined using a rat IgE ELISA set and rat IgA ELISA set, respectively (all from BD Biosciences, Oxford, UK). For evaluation of IgE levels in the BAL, non-diluted BAL samples were used, while a 1:20 dilution was employed for the IgA ELISA in the BAL.

***Histological analysis (H&E, PSR, and PAS staining)***

At each time point, the excised lungs were fixed by inflating with 10% neutral buffered formalin through the trachea and kept overnight. Lung tissues were processed using standard techniques for histological analysis and stained with hematoxylin and eosin (H&E). To quantify the collagen content in the lung tissues, which is a representative indicator for lung fibrosis, picrosirius red (PSR) staining was performed according to the manufacturer’s instructions (Sigma-Aldrich). For detection of goblet cells which contain mucin, periodic acid-Schiff (PAS) (Sigma-Aldrich) staining was performed according to standard methods. The quantitative image analysis of PAS-positive cells was performed using Image-Pro Plus (Media Cybernetics, MD, USA). PAS-positive signals in the airways were separately evaluated according to airway diameter; airways smaller than 1 mm were considered to be small airways or bronchioles and larger than 1 mm were considered to be a large airway or bronchi [5]. The total area of PAS-positive cells was divided by the total area of epithelial cells including basement membrane. The data were expressed as percentage of PAS-positive area versus total epithelial area.

***Immunohistochemistry for eotaxin, TGF-β, and α-SMA***

Immunohistochemical staining for eotaxin, transforming growth factor-beta (TGF-β), and alpha-smooth muscle actin (α-SMA) was performed on lung sections. Briefly, paraffin sections were deparaffinised and rehydrated. For antigen retrieval, BORG Decloaker (Biocare Medical, Walnut Creek, CA, USA) was applied for eotaxin; for TGF- β and α-SMA, sections were microwaved in 10mM citrate buffer, pH 6.0 (2 x 10 mins) before washing in TBS. Endogenous peroxidase activity was quenched by incubating with 3% hydrogen peroxide (in water) at room temperature for 15 min. Slides were then blocked with normal goat serum and primary antibodies were applied. Anti-mouse eotaxin antibody (R&D systems) was applied at 1/50 dilution. Rabbit polyclonal anti-TGF-β (Santa Cruz Biotechnology, USA) and rabbit polyclonal anti-α-SMA antibody (Lab Vision, California, USA) were applied at 1 μg/ml and 0.5 μg/ml, respectively. As a control, the same protocol was applied to serial-sectioned slides with omission of the primary antibody in each case. There was no positivity in the case of negative controls. Slides were washed three times and incubated for 30 min at room temperature with ImmPRESS anti-goat IgG kit (Vector Laboratories, Burlingame, CA) for eotaxin and goat anti-rabbit secondary antibody for TGF-β and α-SMA. Slides were washed three times and DAB substrate (Vector Laboratories) was applied. Sections were counterstained with Gill-2 haematoxylin (Thermo Shandon, USA).

***Transmission electron microscopy (TEM)***

TEM was used to evaluate ultra-structural changes in the lungs induced by instillation of ZnONP. Lungs of vehicle control and ZnONP treated rats 4 wks after instillation were fixed with 1.5% glutaraldehyde in 0.1 M cacodylate buffer, stained *en bloc* with uranyl acetate, and embedded in epoxy resin. Ultra-thin (60 nm) sections were cut, stained with uranyl acetate and lead citrate, and examined with a TEM (JEM-1200EX II, JEOL, Tokyo, Japan).

***Instillation of alternative ZnONP***

To evaluate whether the eosinophilic inflammation was induced by specific types of ZnONP, we instilled another type of ZnONP (designated ZnONP_alt_) into rats. ZnONP_alt_ were purchased from Nanostructural and Amorphous Materials, Inc. (Houston, TX, USA) (Table 1). The diameters of the ZnONP_alt_ were 137 ± 9.2 nm. ZnONP_alt_ were instilled into female Wistar rats at 310 µg per rat, which is the same mass of 150 cm^2^ per rat as the ZnONP from NanoScale Corporation. Four rats were used for each treatment group. After 24 h, rats were euthanized and BAL collection and analysis was performed as described above.

***Instillation of ZnONP-free BAL extract to rats***

To evaluate whether inflammatory mediators produced by ZnONP instillation can produce similar pathologies to that seen with ZnONP treatment, we extracted ZnONP-free BAL fluid and instilled this in rats. Briefly, female Wistar rats were instilled with ZnONP at 150 cm^2^ per rat and BAL was extracted by lavaging with 4 ml of saline. BAL fluid was immediately centrifuged at 250 × *g* for 5 min and cell-free supernatants were collected. Then ZnONP-free supernatants were collected by three rounds of centrifugation at 13 000 × *g*. Then, 500 µl of ZnONP-free BAL fluid was instilled to new female Wistar rats and cytological and histological analysis was performed at 1 and 4 wks after instillation as described above. Five rats (four for BAL analysis and one for histology) were used for each group.

***Instillation of dissolved Zn^2+^ to rats***

To evaluate the effects of dissolved Zn^2+^ in the acidic solution, 1 mg/ml of ZnONP in HCl-acidified saline were dissolved at a pH of 4.5. After 1 wk, ZnONP-free supernatant was collected by three rounds of centrifugation at 13000 × *g*. The supernatant was filtered three times through a 0.22 µm filter (Millipore, Cork, Ireland) to exclude possible bacterial contamination. The concentration of Zn^2+^ of supernatant was measured by inductively coupled plasma-atomic emission spectrometry (ICP-OES) (Perkin Elmer Optima 5300 DV ICP-OES). The pH of the dissolved Zn^2+^ was 6.5, which was less acidic than 0.9% saline (pH 5.5). Thereafter 92.5 and 277.5 µg of Zn^2+^ were instilled intratracheally into rats and cytological and histological evaluation was performed at 24 h and 4 wks after instillation.

***Aspiration of ZnONP into mice***

To evaluate whether the eosinophilic inflammation was a species- and strain-specific phenomenon, ZnONP were aspirated into C57BL/6 and BALB/c mice. ZnONP were dispersed in 5% heat-inactivated mouse serum (collected from healthy C57BL/6 mice) to a concentration of 150cm^2^/ml and 100 µl (15 cm^2^ ZnONP) aspirated into the lungs of each mouse, which were sacrificed 24 h later. As a control, NiONP known to cause acute neutrophilic inflammation [6] were aspirated at the same surface area dose. The characterization of NiONP is presented in Table 1. Saline with 5% mouse serum was aspirated at the same volume as a vehicle control. Four mice per each group were used for cytological evaluation. BAL fluid was extracted by lavaging with 0.8 ml of saline with three times. Preparation of cytospin slides was then performed and analyzed as described above. Eotaxin (R&D systems) and IL-13 (Invitrogen) levels were measured in the BAL samples as described above. TEM was also applied to evaluate the ultra-structural changes in the lung with the same method described above.

***Acridine orange staining***

To evaluate the lysosomal membrane destabilization by NP, acridine orange staining was applied to THP-1 cells. Human monocytic cell line THP-1 was obtained from American Type Culture Collection (ATCC) and cultured at 37 °C with 5% CO_2_ in RPMI containing 10% FBS, 2 mM L-glutamine (Life Technologies, Paisley, UK), 100 IU/ml penicillin, and 100 U/ml streptomycin (Life Technologies). THP-1 cells (1 × 10^6^ cells/ml) were seeded to a µ-Dish^35mm, high^ (Thistle Scientific Ltd., Glasgow, UK) and differentiated using 10 ng/ml of phorbol myristate acetate (PMA; Sigma-Aldrich) for 48 h. After activation, cells were washed three times with PBS and stained with 5 µg/ml acridine orange (Sigma-Aldrich) for 15 min. Cells were then washed three times with PBS and were treated with ZnONP (10 cm^2^/ml; 20 µg/ml) for 24 h. As a control, TiO_2_NP (10 cm^2^/ml; 36 µg/ml) were used. Cells were examined and photographed in a Leica SP5 confocal microscope (Leica Microsystems, Buckinghamshire, UK). For evaluation of cytotoxicity of NP, cells (1 × 10^6^ cells/ml) were cultured in 24-well plate and same process was applied as mentioned above. The supernatants of NP-treated cells were collected and centrifuged for 30 min at 13 000 × *g* to remove the NP. Cytotoxicity of NP-free supernatants was then measured using a lactated dehydrogenase assay kit according to the manufacturer’s manual (Roche Diagnostics Ltd.). Vehicle control and 0.1% Triton-X treatment were used as a negative and positive control, respectively. The cytotoxicity of NP was expressed by percentage of complete cytotoxicity (0.1% Triton-X treatment).

***Statistical analysis***

Data are expressed as mean ± S.D. and were analyzed with GraphPad InStat software (Version 3, GraphPad Software, Inc., La Jolla, CA). To compare each treatment group, one-way analysis of variance with Tukey’s post hoc pairwise comparisons was applied. Student t-test was applied for comparison between vehicle control and ZnONP treatment group in C57BL/6 and BALB/c mice or ZnONP_alt_ treatment group. We considered *p* < 0.05 to be statistically significant.

**References**

1. Stopford W, Turner J, Cappellini D, Brock T: **Bioaccessibility testing of cobalt compounds.** *J Environ Monit* 2003, **5:**675-680.

2. Moss OR: **Simulants of lung interstitial fluid.** *Health Phys* 1979, **36:**447-448.

3. Stebounova L, Guio E, Grassian V: **Silver nanoparticles in simulated biological media: a study of aggregation, sedimentation, and dissolution.** *Journal of Nanoparticle Research* 2011, **13:**233-244.

4. Duffin R, Tran L, Brown D, Stone V, Donaldson K: **Proinflammogenic effects of low-toxicity and metal nanoparticles in vivo and in vitro: highlighting the role of particle surface area and surface reactivity.** *Inhal Toxicol* 2007, **19:**849-856.

5. Siddiqui S, Jo T, Tamaoka M, Shalaby KH, Ghezzo H, Bernabeu M, Martin JG: **Sites of allergic airway smooth muscle remodeling and hyperresponsiveness are not associated in the rat.** *J Appl Physiol* 2010, **109:** 1170-1178.

6. Cho WS, Duffin R, Poland CA, Howie SE, Macnee W, Bradley M, Megson IL, Donaldson K: **Metal Oxide Nanoparticles Induce Unique Inflammatory Footprints in the Lung; Important Implications for Nanoparticle Testing.** *Environ Health Perspect* 2010, **118:**1699-1706.
