# Supplementary figures and images for: Progressive severe lung injury by zinc oxide nanoparticles; the role of Zn2+ dissolution inside lysosomes
Source: Part Fibre Toxicol. 2011 Sep 6;8:27. doi: 10.1186/1743-8977-8-27 (PMC3179432; doi:10.1186/1743-8977-8-27)

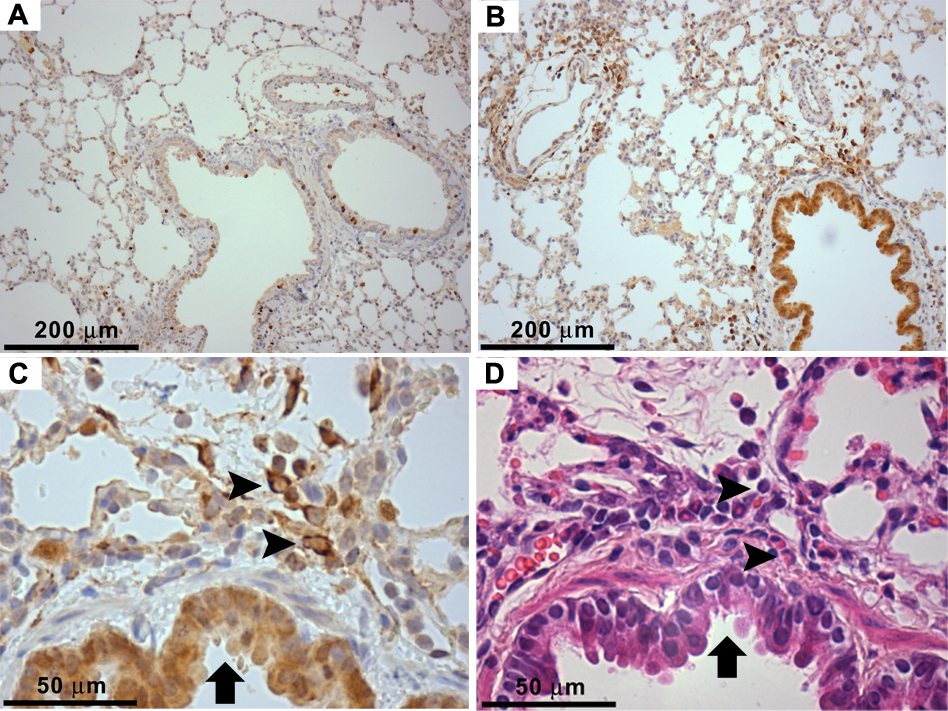

Supplement: Additional file 2 — Immunohistochemistry for eotaxin in the lung tissues 24 h after instillation of ZnONP at 150 cm2/rat. (A), vehicle control; (B and C), ZnONP treatment; (D) serially sectioned H&E staining of (C). (B) is imaged under higher magnification at 400× in (C). Note that eotaxin was strongly positive in the bronchial epithelial cells (arrow) and inflammatory cells (arrowhead). [file 1743-8977-8-27-S2.DOC]

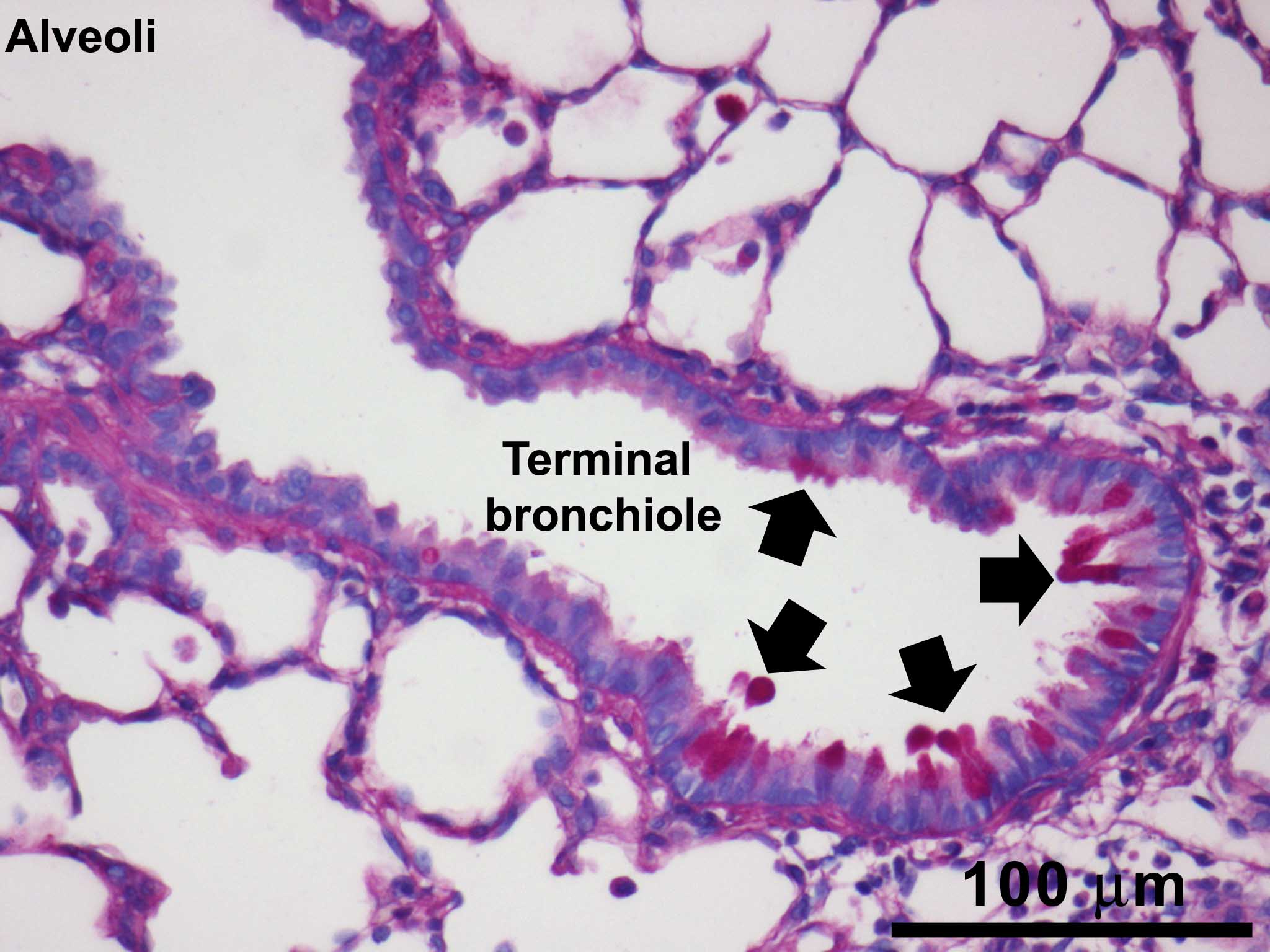

Supplement: Additional file 3 — PAS staining for goblet cells in the lung tissues 1 wk after instillation of ZnONP at 150 cm2 per rat. Goblet cells were found even in terminal bronchioles (arrow) but were not present in the transitional region between bronchiolar and alveolar tissue. [file 1743-8977-8-27-S3.DOC]

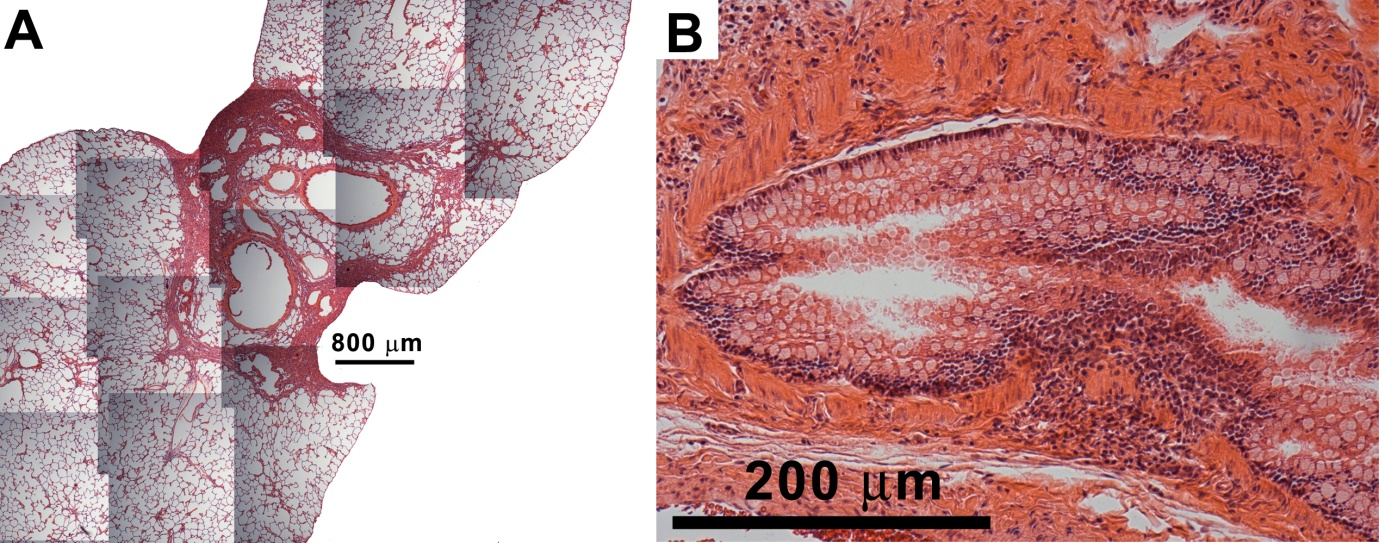

Supplement: Additional file 5 — Representative lung lesion 4 wks after instillation of Zn(II) at 92.5 μg per rat. (A) The lungs showed fibrosis, contraction, atelectasis, and (B) goblet cell hyperplasia. [file 1743-8977-8-27-S5.DOC]

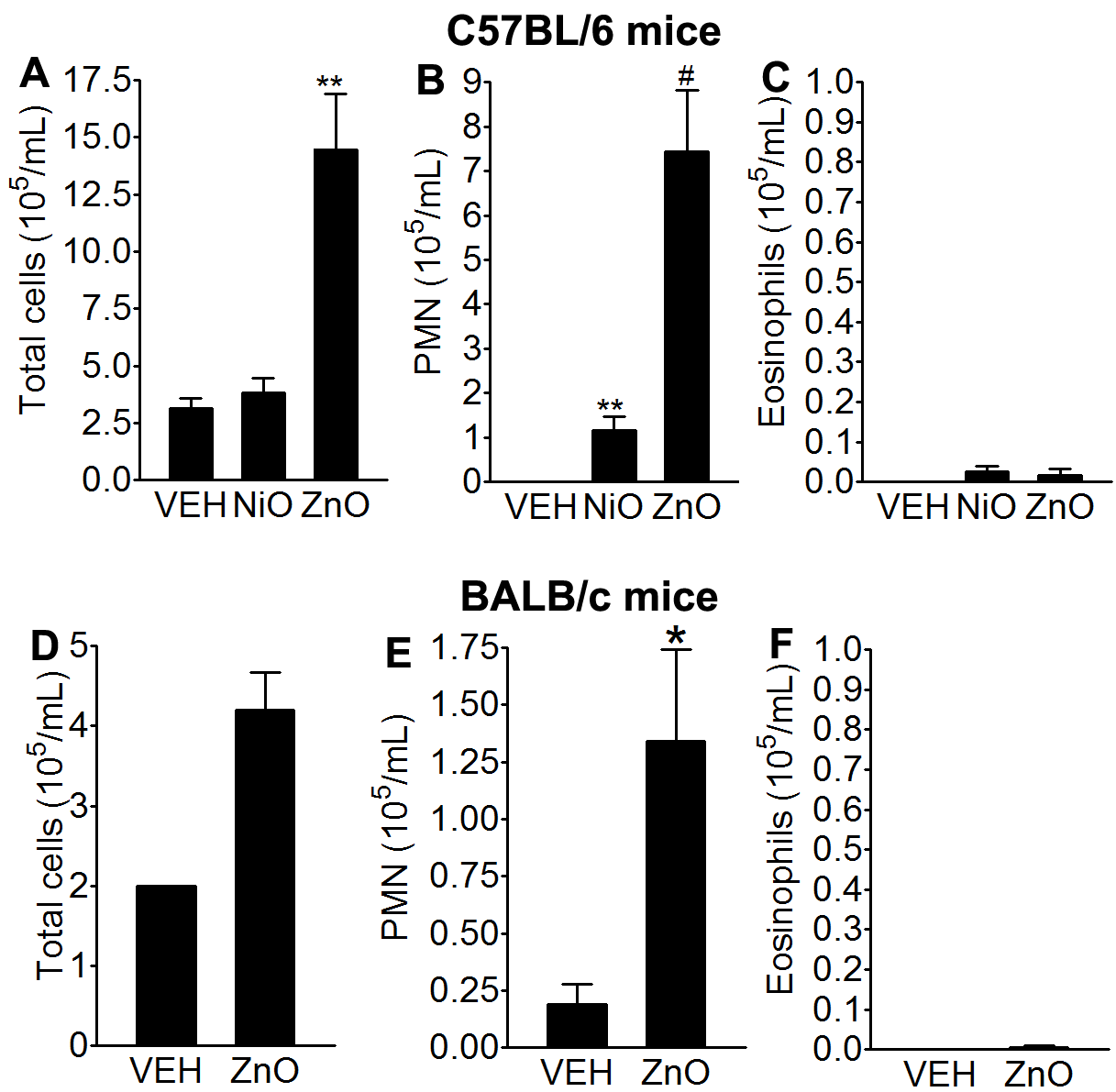

Supplement: Additional file 6 — Pulmonary toxicity of ZnONP at 24 h after aspiration into lungs of C57BL/6 (A - C) or BALB/c (D - F) mice. (A, D), number of total cells; (B, E), number of PMN; (C, F), number of eosinophils. Values are mean ± S.D. n = 4 for each treatment group. Significance versus vehicle control (VEH): * p < 0.05, ** p < 0.01, # p < 0.001. [file 1743-8977-8-27-S6.DOC]

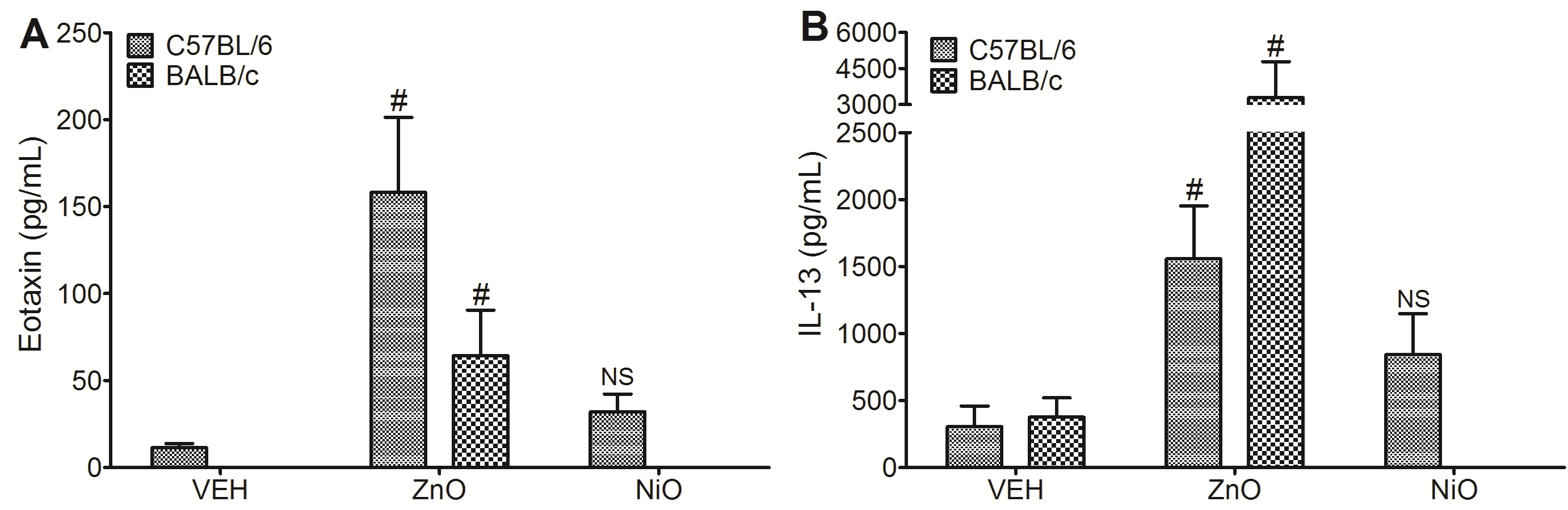

Supplement: Additional file 7 — Expression of eotaxin and IL-13 in the BAL from mice 24 h after aspiration of ZnONP or NiONP at 15 cm2 per mouse. (A), eotaxin; (B), IL-13. Values are mean ± S.D. n = 4 for each treatment group. Significance versus vehicle control (VEH): * p < 0.05, ** p < 0.01, # p < 0.001. NS, not significant. [file 1743-8977-8-27-S7.DOC]

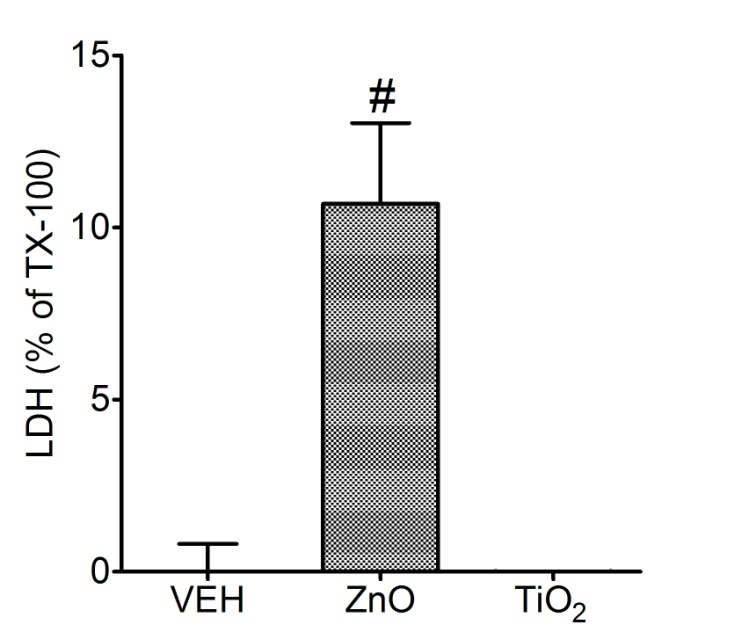

Supplement: Additional file 8 — Cytotoxicity of THP-1 cells after exposure to NP for 24 h, measured as percentage compared to complete lysis (Triton X-100). THP-1 cells were differentiated by treatment with PMA (10 ng/ml) for 48 h and LDH levels were measured 24 h after NP treatment. Values are mean ± S.D. n = 4 for each treatment group. Significance versus vehicle control (VEH): # p < 0.001. [file 1743-8977-8-27-S8.DOC]
