# Supplementary material for: Progressive severe lung injury by zinc oxide nanoparticles; the role of Zn2+ dissolution inside lysosomes
Source: Part Fibre Toxicol. 2011 Sep 6;8:27. doi: 10.1186/1743-8977-8-27 (PMC3179432; doi:10.1186/1743-8977-8-27)
Supplement: Additional file 4 — The effects of hydrodynamic size of ZnONP on the eosinophilia (n = 4). This file contains the number of eosinophils in the BAL after instillation of well-dispersed or highly agglomerated ZnONP. [file 1743-8977-8-27-S4.DOC]

The effects of hydrodynamic size of ZnONP on the eosinophilia (*n* = 4).

| Group^a^ | Hydrodynamic size (nm) | Number of eosinophils (×10^5^) | Percentage of eosinophils in the BAL |
| --- | --- | --- | --- |
| ZnONP-agglomeration^b^ | 4380 | 0.91 ± 0.3 | 1.3 ± 1.2 |
| ZnONP-dispersion^c^ | 242.9 | 5.95 ± 2.7 | 36.7 ± 14.7 |

^a^Female Wistar rats were intratracheally instilled at 150 cm^2^ per rat and BAL fluid analysis was performed 24 h after treatment.

^b^ZnONP were dispersed in the saline without any dispersant.

^c^ZnONP were dispersed with 5% rat serum in saline.
